# Experimental study, simulation and technical–economic feasibility of an interesterification plant for hydrocarbons synthesis by using plastics and frying oil waste

**DOI:** 10.1038/s41598-024-60851-8

**Published:** 2024-05-03

**Authors:** Hugo Gomes D’Amato Villardi, Madson M. Nascimento, Fernando Luiz P. Pessoa, Alex Álisson B. Santos, Luiz Alberto Brêda Mascarenhas, Leone Peter Correia Andrade, Jailson B. de Andrade

**Affiliations:** 1 Centro Universitário SENAI-CIMATEC, Av. Orlando Gomes, 1845-Piatã, Salvador, BA 41650-010 Brazil; 2 Instituto Nacional de Ciência e Tecnologia em Energia e Ambiente-INCT E&A, Universidade Federal da Bahia, Salvador, BA 40170-115 Brazil; 3 Centro Interdisciplinar de Energia e Ambiente-CIEnAm, Universidade Federal da Bahia, Salvador, BA 40170-115 Brazil; 4 Postgraduate Program in Computer Modeling and Industrial Technology, Salvador, BA, Brazil

**Keywords:** Hydrocarbons, Raw materials, Interesterification, Experimental, Simulation, Economic analysis, Chemistry, Energy science and technology

## Abstract

This work presents the experimental assessment of a 20 mL batch reactor’s efficacy in converting plastic and oil residues into biofuels. The reactor, designed for ease of use, is heated using a metallic system. The experiments explore plastic solubilization at various temperatures and residence times, employing a mixture of distilled water and ethylene glycol as the solvent. Initial findings reveal that plastic solubilization requires a temperature of 350 °C with an ethylene glycol mole fraction of 0.35, whereas 250 °C suffices with a mole fraction of 0.58. Additionally, the study includes a process simulation of a plant utilizing a double fluidized bed gasifier and an economic evaluation of the interesterification/pyrolysis plant. Simulation results support project feasibility, estimating a total investment cost of approximately $12.99 million and annual operating expenses of around $17.98 million, with a projected payback period of about 5 years.

## Introduction

The energy requirements of contemporary societies are substantial, with diverse forms of energy essential for a wide array of daily activities. Globally, there is a forecasted one-third surge in energy demand from 2015 to 2040, predominantly attributed to nations beyond the purview of the Organization for Economic Cooperation and Development^1^.

The primary drivers behind this escalating demand encompass several key factors: the anticipated global population surge from 7 billion presently to 9 billion by 2040, alongside a projected 3.6% annual expansion in the world economy over the corresponding period, in conjunction with discernible trends towards heightened urbanization and mobility^2,3^. As household consumption patterns elevate, industrial sectors grow, and urbanization intensifies, there has been a concurrent surge in fuel consumption, accompanied by a proliferation of solid waste pollution^4^. Among the myriad waste products, plastics emerge as a prominent component.

Plastic, a synthetic polymer derived from petroleum, encompasses organic components, and boasts remarkable durability, rendering it a ubiquitous raw material for the fabrication of diverse objects. To illustrate the scale, global plastic production reached approximately 335 million tons in 2020^5^. However, this surge in production translates into a substantial volume of plastic waste, notably including plastic bags and PET bottles, which find their way into marine ecosystems, posing significant environmental challenges. A study conducted by the UK government^6^ revealed a distressing projection: by 2025, the pollution levels of plastic in the world's oceans are expected to triple. Moreover, current estimates suggest that the oceans are already burdened with a staggering minimum of 5.25 trillion plastic pieces, averaging five millimeters in size. Another study forewarns that by 2050, the quantity of plastic pieces in the oceans will surpass that of fish^7^. Additionally, approximately eight million tons of plastic waste and its derivatives are discharged into the oceans annually^8^.

Despite widespread campaigns advocating for plastic recycling and even advocating for the complete elimination of certain plastic products, the persistence of unrecyclable plastic waste remains a significant concern. However, recent research focusing on converting plastic waste into fuels has emerged as a sustainable alternative that addresses both plastic recycling and renewable energy production. Techniques such as pyrolysis, catalysis, and interesterification reactions have garnered attention as the most frequently reported methods for generating gasoline or diesel-range hydrocarbons from plastic^9–11^.

In addition to plastics, another globally pervasive environmental waste is frying oil. A significant contributor to this type of liquid waste is vegetable oil, extensively utilized in industrialized nations^12^. Current estimates indicate global vegetable oil consumption exceeding 200 million metric tons^12,13^. Consequently, vast quantities of liquid waste are generated, often disposed of improperly in sewers, municipal waste systems, open areas, rivers, and even oceans in many urban centers^12^. Improper disposal of frying oil waste yields several environmental impacts, including diminished concentrations of dissolved organic oxygen in aquatic ecosystems, formation of foam, and contamination of freshwater sources^12^. While some efforts have been made to repurpose frying oil waste into biofuels such as biodiesel^14,15^, there remains a dearth of published research proposing the utilization of a blend of plastic and frying oil waste for biofuel production.

The utilization of thermochemical treatment methods, such as pyrolysis, has traditionally served as the primary approach for converting solid waste into fuels^16–19^. Recent studies have highlighted the potential of co-pyrolysis involving a combination of waste plastics and biomass, in producing high-value products like bio-oil, bio-coal, and syngas^8,11,20–22^. These experiments are typically conducted in fixed bed pyrolyzers. Optimal outcomes, particularly in bio-oil yield (62.57%), have been observed at a temperature of 400 °C with an initial plastic content of 70%^20^. Conversely, the prevalent pathway for converting vegetable oil waste into biofuels involves alkaline interesterification reactions. However, our previous research has demonstrated that fatty acids derived from vegetable oil can be efficiently transformed into esters utilizing a batch reactor operating at elevated temperatures^23^.

Based on this rationale, we propose that a feasible approach for converting a combination of solid plastic and liquid frying oil wastes involves employing a technique capable of yielding a product with enhanced added value, such as supercritical synthesis, which can generate glycerin devoid of contaminants or through interesterification with methyl acetate. This process occurs in a single step, wherein the methyl acetate reacts with the oil's triglycerides to produce fatty acid methyl esters (FAME) and triacetin as a byproduct. Should free fatty acids be present in the oil, they undergo esterification with methyl acetate to form FAME, with acetic acid generated as a parallel byproduct. The significant advantage of this route lies in the production of triacetin as a byproduct. While triacetin holds a higher commercial value than glycerol, a potentially advantageous strategy for commercial exploitation involves incorporating the volume of byproduct generated into the primary product, thereby yielding a greater volume of biofuel from a mixture of FAME and triacetin. Consequently, there would be no necessity to purify triacetin during the process^24^.

Based on the information provided, the recent study aims to ascertain the optimal conditions for synthesizing bio-oil through the interesterification of plastics in the presence of frying oils. The experimental data will be utilized to simulate an industrial plant and subsequently evaluate its economic potential.

## Methods

### Experimental methodology

The experimental methodology commenced with an initial assessment of temperature, followed by sequential evaluations with variable time intervals. Temperature settings were determined based on the constituents’ critical points. The reagents such as ethylene glycol, and ethyl acetate were acquired from Merck (Darmstadt, Germany), and exhibited a purity of 99.9%. Plastic and oil residues were procured from the SENAI CIMATEC restaurant. A 20 mL batch reactor, identical to the one utilized by Villard et al.^23^, was employed to assess the conversion potential of plastic and oil residues into biofuels. The reactor, designed for enhanced handling and mobility, features a metallic heating system with resistances. Reactions occur atop a support structure situated above a thermostatic bath, maintained at – 3 °C using a mixture of distilled water and ethylene glycol. The apparatus includes an 18 mm inlet for raw material supply, alongside two 1/16-inch inlets—one for temperature control and the other connected to a syringe pump (Teledyne ISCO 260 D) facilitating gas and liquid injection under desired conditions of temperature and pressure, while also enabling pressure measurement within the medium. In the experiments, an occupancy level close to 80% of the reactor’s total volume was chosen as a safety precaution due to the lack of pressure control resulting from the incompressibility of larger volume mixtures. The experimental plan, informed by literature^8,17^, encompasses defined variables to elucidate the impact of oil/plastic mixture composition on yield, temperature, and duration. Given the absence of analogous experimental data, preliminary tests were conducted to assess plastic solubilization, executed in triplicate under the conditions outlined in Table 1.

**Table 1:** Exploratory evaluation planning.

Test	T (°C)	N_2_ initial pressure (bar)	Time (min)	× PET _(mole fraction)_	× TRIG _(mole fraction)_	× EG _(mole fraction)_	× EA _(mole fraction)_
1	250	5	30	0.0200	0.0255	0.5822	0.3723
1.1	350	5	30	0.1298	0.0510	0.3486	0.4707
1.2*	250	5	15	0.1361	0.0482	0.2977	0.5180
1.3*	250	5	30	0.1246	0.0430	0.2970	0.5353
1.4	350	5	5	0.0195	0.0182	0.3787	0.5835
1.5*	250	5	5	0.0216	0.0036	0.5869	0.3879
1.6*	300	5	5	0.1358	0.0492	0.3025	0.5125

*  Not solubilized plastics. The following variables were considered: T(°C), temperature; Polyethylene terephthalate (PET), triglycerides (TRIG), ethylene glycol (EG), ethyl acetate (EA).

The points marked with asterisk (1.2, 1.3, 1.5, and 1.6) did not solubilize the plastic, the others did. The results are related to temperature and percentage of ethylene glycol (EG) in the medium. That is, if the mole fraction of EG is 0.35, a temperature of 350 °C is required for solubilization, whereas if the mole fraction is 0.58, 250 °C is sufficient. Another point observed was the residence time, at 250 °C more than 5 min are required for the solubilization of the plastic, as can be observed in the comparison between point 1 and 1.5. At 350ºC this same time is already sufficient for solubilization.

For a more complete understanding, studies will be conducted with both compositions and temperatures, varying the time from 5 to 60 min. The experimental designs for the tests can be seen in Tables 2 and 3.

**Table 2:** Interesterification tests at 350 °C planning.

Test	T (°C)	N_2_ initial pressure (bar)	Time (min)	× PET _(mole fraction)_	× TRIG _(mole fraction)_	× EG _(mole fraction)_	× EA _(mole fraction)_
2.1	350	5	5	0.14	0.05	0.30	0.50
2.2	350	5	15	0.14	0.05	0.29	0.51
2.3	350	5	30	0.14	0.05	0.30	0.51
2.4	350	5	45	0.14	0.05	0.30	0.51
2.5	350	5	60	0.14	0.05	0.30	0.51

The following variables were considered: T(°C), temperature; Polyethylene terephthalate (PET), triglycerides (TRIG), ethylene glycol (EG), ethyl acetate (EA).

**Table 3:** Interesterification tests at 250 °C planning.

Test	T (°C)	N_2_ initial pressure (bar)	Time (min)	× PET _(mole fraction)_	× TRIG _(mole fraction)_	× EG _(mole fraction)_	× EA _(mole fraction)_
3.1	250	5	5	0.02	0.03	0.58	0.37
3.2	250	5	15	0.02	0.03	0.58	0.37
3.3	250	5	30	0.02	0.03	0.58	0.38
3.4	250	5	45	0.02	0.03	0.59	0.37
3.5	250	5	60	0.02	0.03	0.58	0.37

The following variables were considered: T(°C), temperature; Polyethylene terephthalate (PET), triglycerides (TRIG), ethylene glycol (EG), ethyl acetate (EA).

### Composition analysis and chemical characterization

The reagents used were plastic waste, oily waste, ethylene glycol, and ethyl acetate. The operational action elements are temperature and chemical reactions. The tests were carried out in a 20 mL batch reactor, the same one used by Villard et al.^23^. The development of new fuels, preferably those that preserve the environment, by decarbonizing the process or using waste that would otherwise be discarded, is of great importance to the consumer market. However, it is necessary that the developed product be accepted due to its compositional characteristics, and in this specific case, due to its functional and differentiated characteristics compared to the state of the art. Thus, the physicochemical characterization of the developed product was carried out.

The chemical characterization of reaction products was performed by static headspace technique according to the methodology published by Araujo et al.^25^, with modifications. A volume of 2 mL of the sample was transferred to a 20 mL headspace vial previously filled with 8 mL of ultrapure water. The headspace vial was closed using a lid with silicone septum and placed on a heating plate at 75 °C for 5 min coupled to an aluminum device specially designed to keep the temperature homogenous^26^. After that, the analytes were manually injected into a GC–MS QP2010-SE (Kyoto, Japan), using a 2.5 mL Hamilton gastight syringe. The GC–MS was equipped with an Agilent J&W DB-1 column (30 m × 0.25 mm × 0.25 µm), containing a stationary phase composed of 100% dimethylpolysiloxane (Santa Clara, California, USA). The separation and detection of the compounds was carried out according to the following experimental conditions: injector temperature of 250 °C (splitless mode), ultra-pure helium flow (99.999%) at 1.00 mL/min, and temperature programming elution mode, where the initial oven temperature was 35 °C, kept at this temperature for 2 min, then the temperature was increased to 250 °C at a heating rate of 10 °C/min, holding in this temperature for 5 min (total runtime 28.5 min). The mass spectrometer was operated in electron ionization mode at 70 eV. The ion source and interface were kept at 250 °C. The data acquisition was carried out in Full Scan mode, covering a mass range from 45 to 300 *a.m.u*. The compounds identification was based on the mass spectrum fragmentation pattern and comparation with NIST 11 Spectral Library database.

### Simulation methodology

The mean objective is simulating a pilot plant to produce gasoline, ethylene and methane. The simulation was formulated utilizing experimental data, yielding results with less than 5% deviation.

### Thermodynamic model

The thermodynamic package selected was the Peng-Robinson cubic equation of state with Boston-Mathias alpha function, which models the gas phase at medium and high pressures with good accuracy^27^.

### Raw material state

The residual raw material used will be PET and frying oil, since they are major polluters of the environment, mainly water. In the Aspen Plus® v12.1 simulator there is the PET component and its data, without the need to assemble structures and calculate specific properties. For soybean oil, the triglyceride triolein with 5% oleic acid was considered. Ethyl acetate, ethylene, gasoline, and ethyl oleate were present in ASPEN data bank. According to WWF, Brazil alone produces 11,355,220 million tons of plastic waste per year and 1 billion liters of oil are incorrectly disposed of. In the country, there are cooperatives that recycle waste and can be partners in the business^28^.

### Process simulation of a plant

The methodology used was a simulated RStoich reactor in Aspen Plus® v12.1 operating at^27^ 350 °C and 20 bar with products conversions. The raw materials for feeding the gasifier were residual plastic (14 kmol/h), residual soybean oil (5 kmol/h), ethyl acetate (51 kmol/h) and ethylene glycol (30 kmol/h) where total inlet is 100 kmol/h. The criterion adopted for the interesterification reactions was conversion reactor since there is no kinetic data on the proposed route in the literature. Therefore, this reactor was chosen, and the product outputs were tied based on the BR10202301620 patent request. For the synthesis of the final products, equilibrium reactors were used. The premises considered in this work were: 1—steady state and isothermal operation; 2—pressure and temperature are uniform in each reactor; 3—load and heat losses are neglected; 4—drying and pyrolysis stage are instantaneous; 5—ash is inert; 6—each raw material is converted to product as specific efficiency; 7—purification steps represented by numerical separators, because Aspen Plus does not have PSA, so the separation percentage is used in this equipment. However, sizing, CAPEX and OPEX are calculated separately, as will be discussed later.

**Figure 1:**
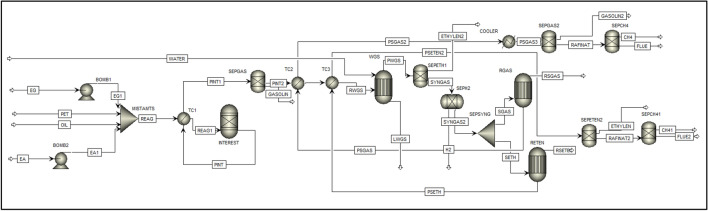
Flowchart of interesterification and pyrolysis from plastics and vegetable oils.

The illustrative scheme of the process simulation is presented in Fig. 1. The MISTAMTS stream is the mixed raw material that, after passing through the exchanger (TC1), feeds the interesterification reactor. The PINT stream that leaves the reactor goes to the TC1 as a hot stream. The output current from the TC1 (PINT1) goes to a separator (SEPGAS), where the syngas is separated from the gasoline. The PINT2 is heated in TC2 e TC3 and goes to water–gas-shift reactor (WGS). The ethylene present in products (PWGS) is separated in SEPETEN, the syngas goes to SEPH2 where the H_2_ product leaves the process. In SEPSYNG, the syngas is separated and is sent to RGAS ^23^, where gasoline is produced, the stream PSGAS is hot stream in TC2 and goes to SEPGAS2, the raffinate product goes to SEPCH4 where CH_4_ is separated. The other outlet stream from SEPSYNG is sent to RETEN^29^ where PSETEN is hot stream about TC3 and follow to SEPETEN2, the raffinate goes to SEPCH41, where the second outlet CH_4_ product. The simulation was made with all consumption of hydrogen because the hydrogen storage is safe less than another product.

### Economic evaluation of the interesterification plant

To estimate the capital cost of the pyrolysis unit, the Lang Method^30^ was used. The total investment capital (Total Capital Investment, TCI) is obtained by Eq. (1):

1 $$C_{TCI} = 1,05 f_{L} \sum i\left( {\frac{{PCI_{i} }}{{PCI_{b,i} }}} \right) C_{i}$$

where: C_TCI_ is the total investment cost including working capital; f_L_ is the Lang Factor, which for a plant that processes solids and fluids is equal to 5.9; C_i_ is the cost of each of the sized equipment; PCI_i_ and PCI_b,i_ are the Plant Cost Index in the current year and in the base year, respectively.

The value of the Plant Cost Index (PCI) provided by the Chemical Engineering magazine^30^ for the year on which the correlations were based was 394. For the year 2023, this value is 702.3. The methodology is like that used by^22^.

Using the data obtained in the simulation, the design was based on the equations proposed in the literature^30^. The flow rates (m^3^/h), operating pressure (psig) and temperature (ºF) obtained from the ASPEN simulation data were used as input data. The reactor volume was determined by the quotient between inlet flow and residence time, as well as the catalyst composition.

### Production costs

In addition to equipment investments, it is necessary to calculate the production cost of the plant. From there, the direct and indirect costs of the plant were described, as well as the revenue obtained from the sale of products. Production costs are estimated based on Turton et al.^31^ and an explanatory summary is presented in Table 4.

**Table 4:** Correlations for the calculation of production costs^31^.

Cost	Correlation
Investment	
Working capital	0.15 of Fixed Investment
Direct costs	
Technical supervision	0.18 of Labor
Maintenance and repairs	0.06 of Fixed Investment
Operational supplies	0.15 of Maintenance and Repairs
Laboratory charges	0.15 of Labor
Patents and royalties	0.03 of Production Cost
Indirect costs	
Packaging and storage	0.6 of (Labor + Technical Supervision + Maintenance and Repairs)
Local taxes	0.032 of Fixed Investment
General expenses	
Administrative costs	0.15 of (Labor + Technical Supervision + Maintenance and Repairs)
Distribution and sale of products	0.11 of Production Cost
Research and development	0.05 of Production Cost

A scenario of 50% hydrogen recovery and syngas separation to 50% gasoline and 50% ethylene was considered. The equipment was then dimensioned, and the costs were estimated for that scenario. Production costs were calculated following the methodology of the literature^31^, considering that the plant operates 24 h a day and 334 days a year, totaling 8016 h.

The base prices considered for the raw material, the products, and utilities, in addition to the catalysts, along with their composition and density, were taken from the COMEX STAT (portal for accessing Brazilian foreign trade statistics), obtained by the ratio between the f.o.b import value, in US dollars, and the net import kilogram value for 2023.

The price of electricity and water were obtained from the tariffs published by ANEEL—National Agency of Electric Energy—for the industrial sector, in August 2023 and by ANA—National Agency of Water and Basic Sanitation for the same sector and period, the conversion currency was carried out using the August 2023 quotation according to the Central Bank of Brazil.

The economic indicators evaluated to propose the feasibility of the project were the Net Present Value (NPV), the Internal Rate of Return (IRR), the Profitability Index (PI) and Payback.

## Results and discussion

### Experimental results

The comprehensive characterization of the products derived from the conversion of plastic and frying oil wastes hinges upon chromatographic analysis; however, solubilization outcomes can be visually discerned. Initial runs have been conducted, revealing that at 350 °C, all tests effectively solubilize PET and yield a single phase. Conversely, at 250 °C, a minimum residence time of 30 min is requisite, resulting in the formation of two phases (oil and triacetin) in these instances.

**Figure 2:**
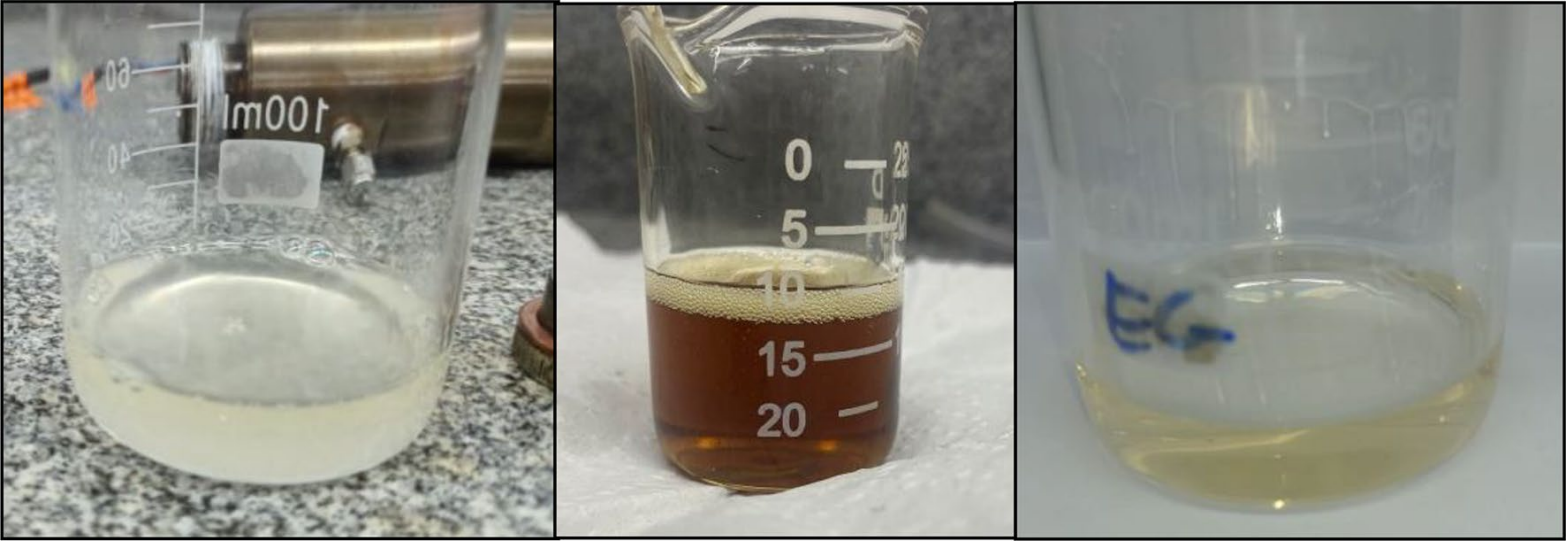
Generated products in tests 1, 1.1 and 1.4 (solubilized PET).

**Figure 3:**
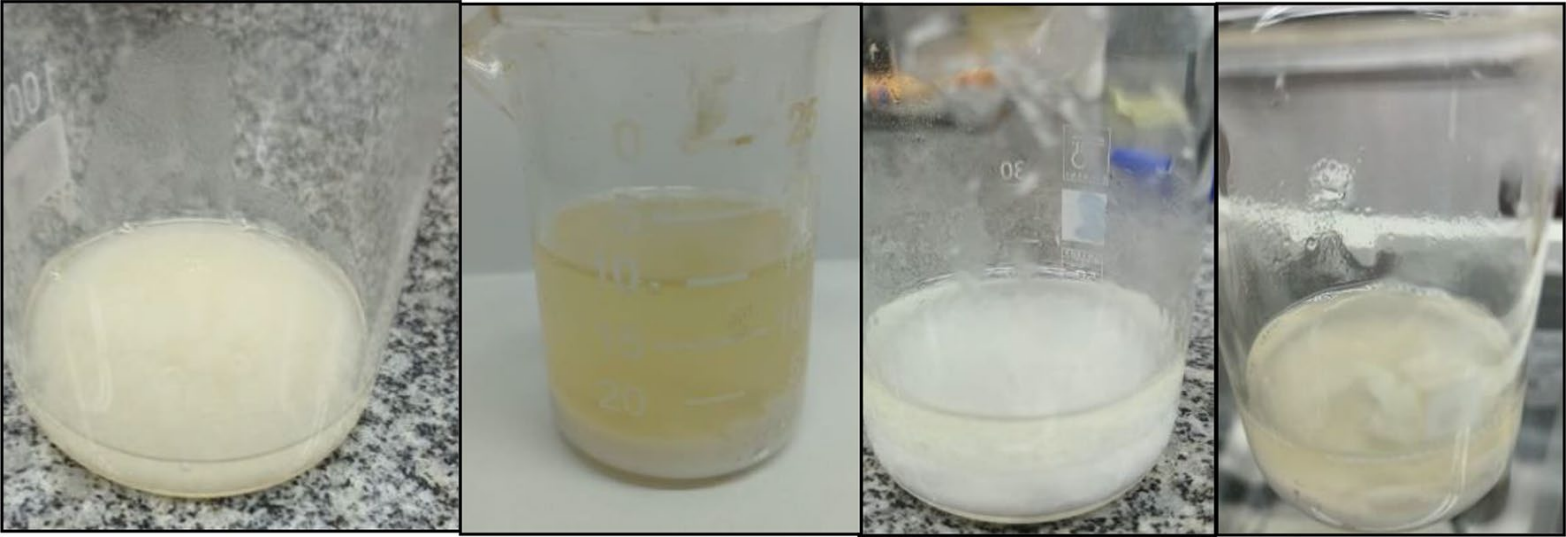
Generated products in tests 1.2, 1.3, 1.5 and 1.6 (no solubilized PET).

Notably, experiments 1.2, 1.3, 1.5, and 1.6 (Table 1) failed to induce plastic solubilization, while the remaining experiments were successful. These outcomes are intricately linked to temperature and the percentage of ethylene glycol (EG) within the medium. Specifically, a mole fraction of EG at 0.35 necessitates a temperature of 350 °C for effective solubilization, whereas a mole fraction of 0.58 suffices at 250 °C. Additionally, the significance of residence time emerges as a noteworthy observation; at 250 °C, a duration exceeding 5 min proves requisite for plastic solubilization, as observed in the comparison between point 0 and 0.5. Conversely, at 350 °C, this same duration is already sufficient for solubilization. Detailed results are depicted in Figs. 2 and 3.

The results show the potential of the technique of plastic and oil conversion to biofuels. However, an additional characterization by gas chromatography-mass spectrometry is essential to fully understand the process efficiency, especially the conversion to BHET, esters and triacetin, besides the fuel range of hydrocarbons, such as aromatics and saturated alkanes. In addition, the data allowed to define the best way to utilize the final product.

**Figure 4:**
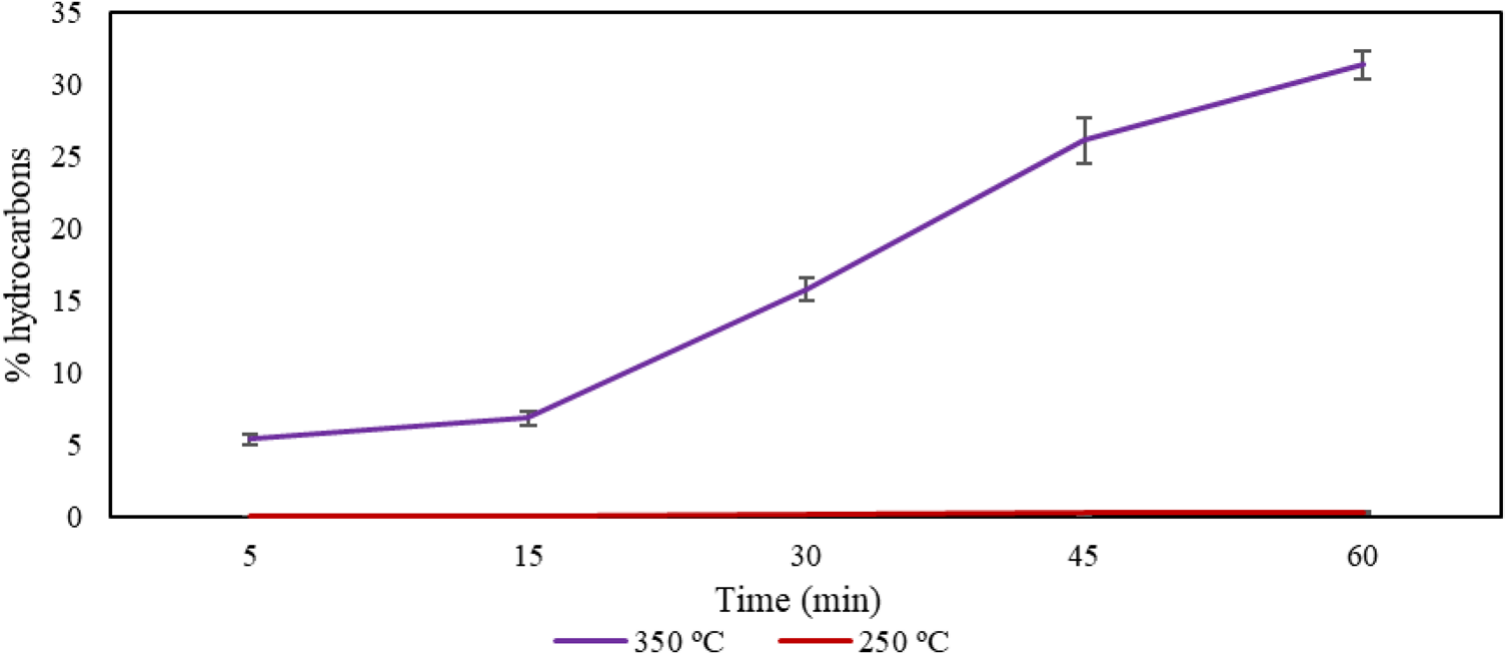
Graph showing the hydrocarbon conversions of the performed tests (average of duplicates).

The results of hydrocarbon conversions per test (average of duplicates) are presented in Figure 4, where it can be observed that the tests at 250 °C showed no conversion. The tests at 350 °C showed increasing values over time, with the 60-min test reaching over 39% of hydrocarbons in the C_8_–C_11_ range.

**Figure 5:**
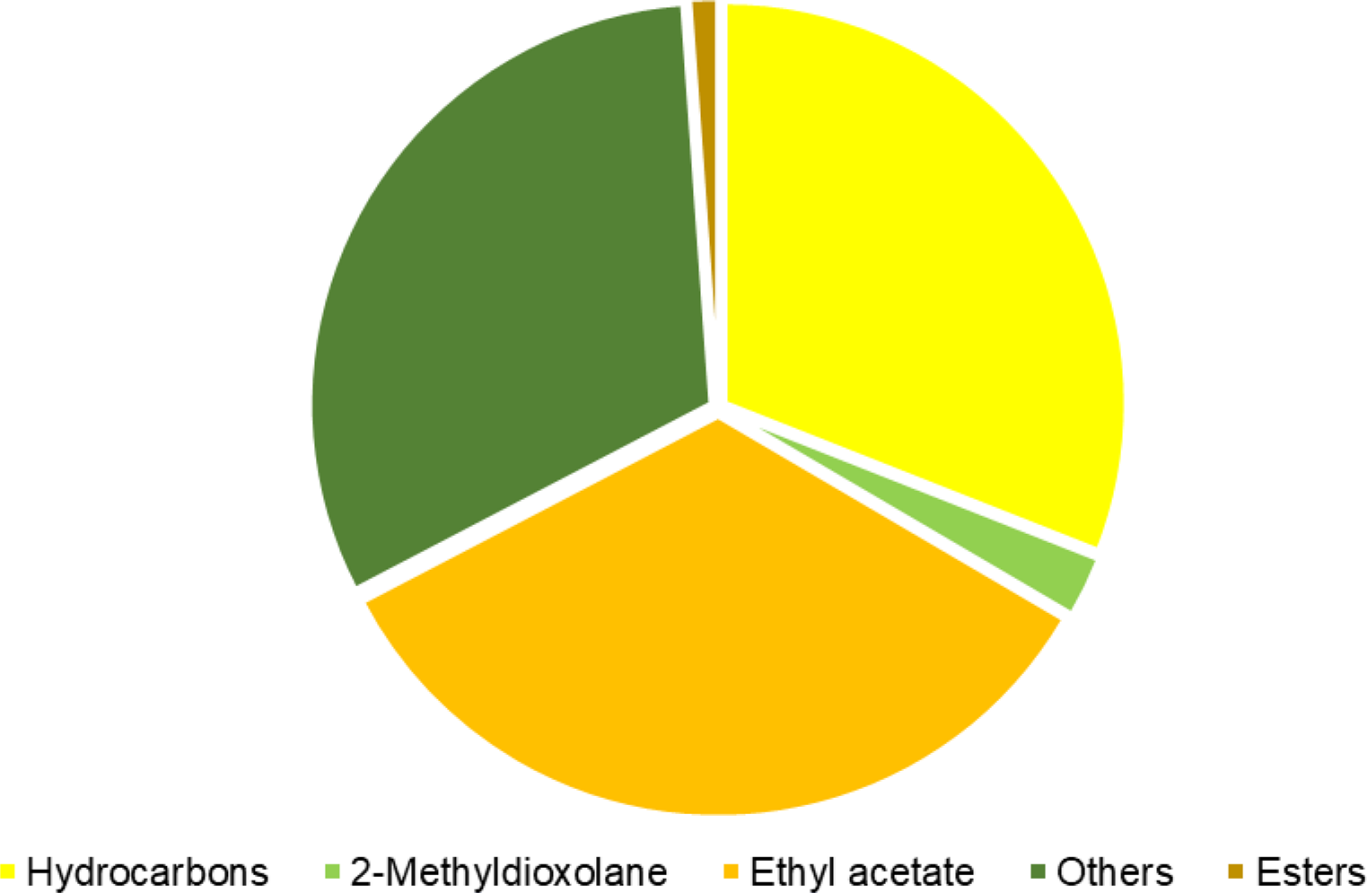
Composition of the product obtained in the test at 350 °C and 60 min.

**Figure 6:**
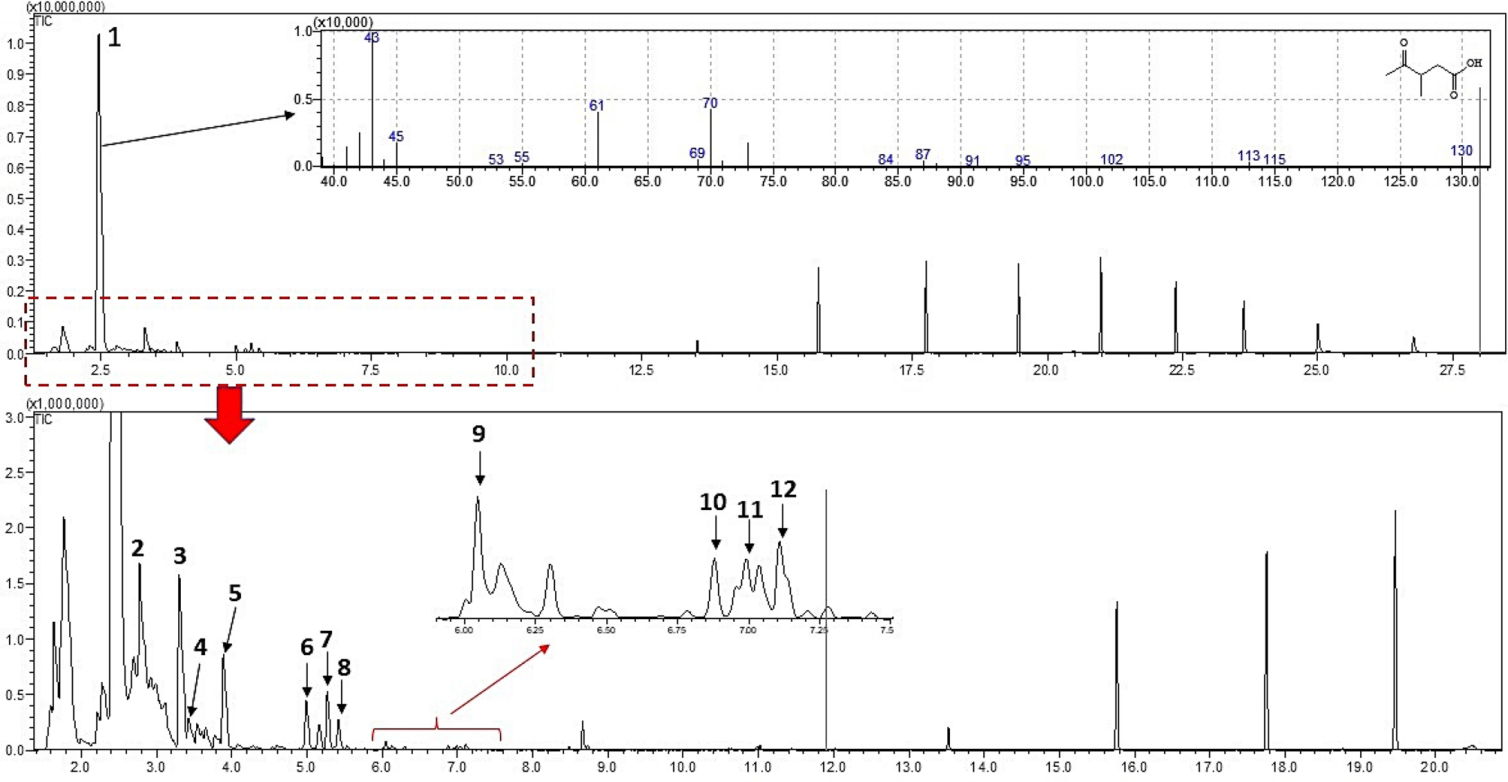
Total ion chromatogram (TIC) of a sample showing the identified compounds and mass spectrum obtained by electron ionization (EI-GC–MS). The compounds are listed in order of elution: [1] ethyl acetate (2.51 min), [2] 1-methyldioxolane (2.77 min), [3] 1-heptene (3.30 min), [4] heptane (3.42 min), [5] 1,1-dioxetane (3.88 min), [6] 1-octene (4.99 min), [7] 2-octene (5.26 min), [8] 4-octene (5.41 min), [9] ethylene glycol monoacetate (6.04 min), [10] 3-nonene (6.99 min), [11] 2,4-dimethylhexane (7.03 min), [12] 2-ethoxyethyl acetate (7.10 min).

Table 5 presents the determined values for the main identified compounds in the sample by GC–MS. In general, the analysis revealed the predominant presence of esters and aliphatic hydrocarbons, including alkanes, alkenes, and cycloalkenes. Most of the reaction products also exhibited the presence of monoaromatic compounds, notably benzene and toluene (See Tables S1–20 in the Supplementary Material). These findings closely align with those reported by Rasul et al.^32^ who conducted a study on the conversion of plastic-based medical wastes into liquid fuel oil. Figure 5 graphically presents the result of the test conducted at 350 °C for 1 h, where the highest percentage of hydrocarbons from the experimental design was observed. Figure 6 shows the chromatogram related to the analysis, indicating the compounds obtained in the biofuel.

**Table 5:** Characterization of the synthesized compound (mean ± standard deviation).

Compounds (%)					Calorific value	
Hydrocarbons	2-Methyldioxolane	Ethyl acetate	Others	Others	(kJ/g)	
30.67 ± 0.01	2.43 ± 0.01	33.56 ± 0.01	1.12 ± 0.01	31.20 ± 0.01	31.5 ± 0.10

### Evaluation economic results

The operational cost (Operational Expenditure—OPEX) corresponds to the cost associated with the daily operation of the pyrolysis plant and its calculation is a function of direct production costs, fixed production costs and general expenses. Among the factors that affect the operational cost, are included: (1) direct costs comprising raw materials, inputs, utilities, labor, preventive and corrective maintenance, operational supplies, etc.; (2) fixed costs comprising depreciation, taxes, plant overheads and (3) overheads comprising cost of administration, distribution and sales and research and development^31^. Table 6 presents the estimates of the operational costs of the pyrolysis plant obtained through the models presented in this work. The total operating cost of the pyrolysis plant was close to US$ 17,983,489.62 per year.

**Table 6:** Annual operating result of the economic evaluation of the pyrolysis plant.

Plant operating costs	
Direct costs	
Raw material (C_RM_)	
Plastic + Oil	US$208,390.00
Labor (C_L_)	US$33,918.16
Technical Supervision (C_TS_)	US$8,479.54
Utilities (C_UTIL_)	
Steam	US$0.00
Cooling water	US$22,896.00
Electricity	US$21,600.00
Catalysts	US$0.00
Adsorbents	US$3,377.80
Effluent disposal (C_ED_)	US$0.00
Maintenance and repairs (C_MR_)	US$119,027.15
Operating supplies (C_OS_)	US$29,756.79
Laboratory charges (C_LAB_)	US$8,479.54
Patents and royalties (C_PR_)	US$22,189.64
Indirect costs	
Packaging and storage (C_PS_)	US$96,854.91
Local taxes (C_LT_)	US$19,837.86
Depreciation (Non-Accounting)	US$198,378.58
General expenses	
Administrative costs (C_ADM_)	US$33,899.22
Distribution and sale of products (C_DSP_)	US$73,965.48
Research and development (C_R&D_)	US$36,982.74
Total operating cost (OPEX)	US$739.654,83/year

The cost of capital (Capital Expenditure—CAPEX) includes investment costs in the pyrolysis plant to become operational and includes direct costs of acquiring equipment, indirect costs, contingency costs, and cost with auxiliary facilities^31^. Table 7 presents the estimates with the investment costs in equipment of the pyrolysis plant together with the indirect and auxiliary costs, added to the working capital necessary to keep the plant in full operation. The total capital cost of the pyrolysis plant was in the order of US$ 2,479,732.29.

**Table 7:** Capital investment result for the pyrolysis plant.

Plant investment cost	
Equipment cost (Separators, gasoline and ethylene reactors, gasifier and heat exchanger)	US$787,216.60
Indirect and ancillary costs	US$1,983,785.84
Working capital	US$1,196,569.23
Total capital cost (CAPEX)	US$2,479,732.29

The prices of the remaining compounds were obtained from COMEXSTAT. The prices per kilogram of each product are presented in Table 8. Hydrogen price will be defined how equilibria parameter; however, the hydrogen will be increased until investment revenue in space time defined.

**Table 8:** Price of products per kilogram.

Product price (US$/kg)	
Gasoline	US$0.53
Ethylene	US$0.35
CH_4_	US$0.20

Table 9 shows the plant's revenue projection data, considering the formation of three main products based on the recovery scenario described in the methodology, without hydrogen.

**Table 9:** Result of the annual revenue projection for the pyrolysis plant.

Plant revenue projection	
Gasoline	US$895,170.00
Ethylene	US$308,700.00
CH_4_	US$559,440.00
Total revenue projection	US$1,203,870.00

### Cashback time

**Figure 7:**
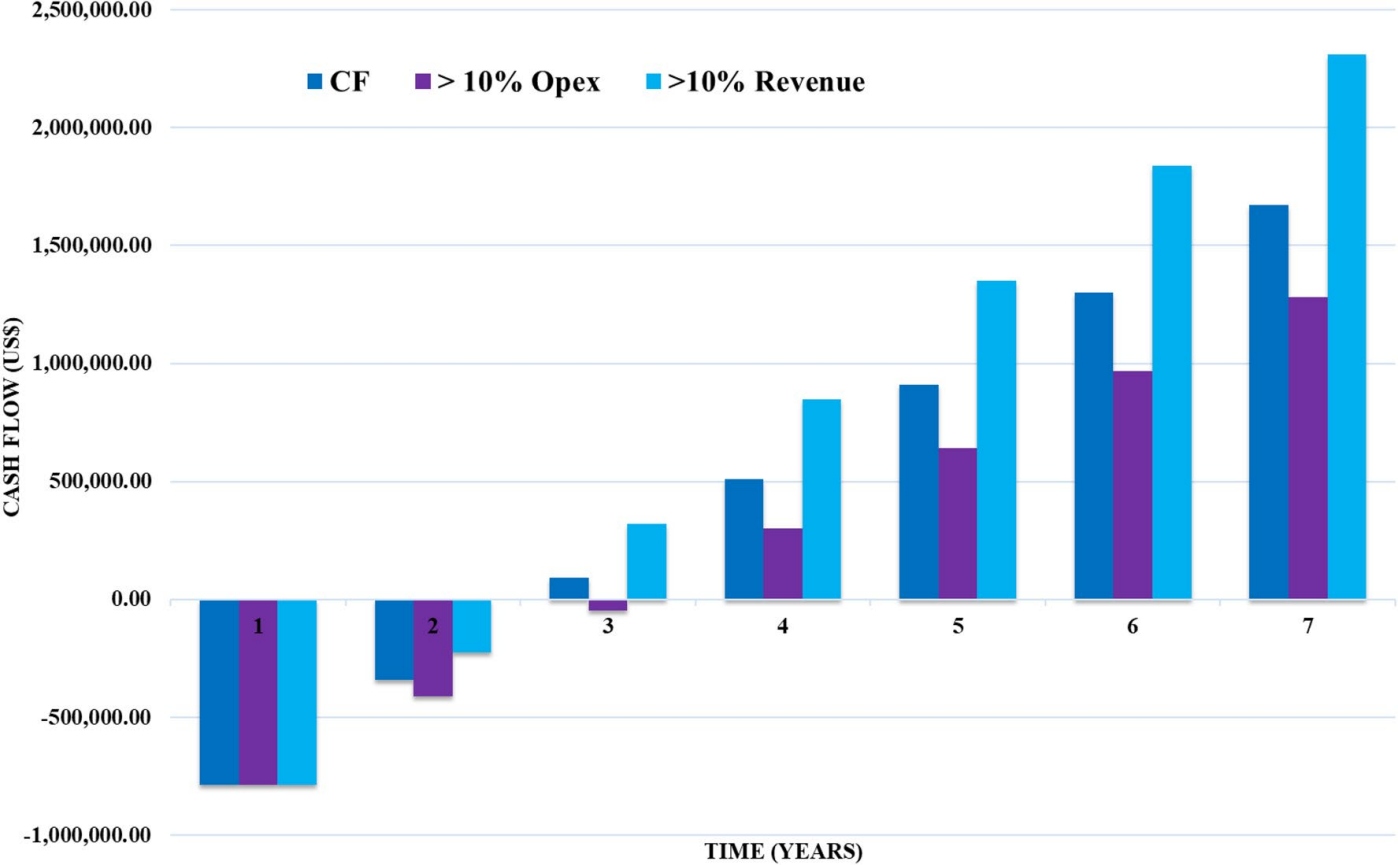
Cash flow with revenue evaluated and 10% increase in OPEX and revenue with company money.

Two cash flow was evaluated, the first was prepared considering the estimated values of OPEX, starting operations in year 1, CAPEX, considering the investment in year zero and the projected revenue in year 1 of the project. To calculate the discounted cash flow (DCF), the net present value (NPV) formula was used, considering a minimum attractiveness rate (MAR) of 11.75% per year and an average inflation rate (IR) of 7.8% in the same period. As can be seen in Fig. 7, was estimated an increase of 10% in OPEX and the same rate to revenue. The results shows than in 4 years, really with OPEX increase the investment returns.

**Figure 8:**
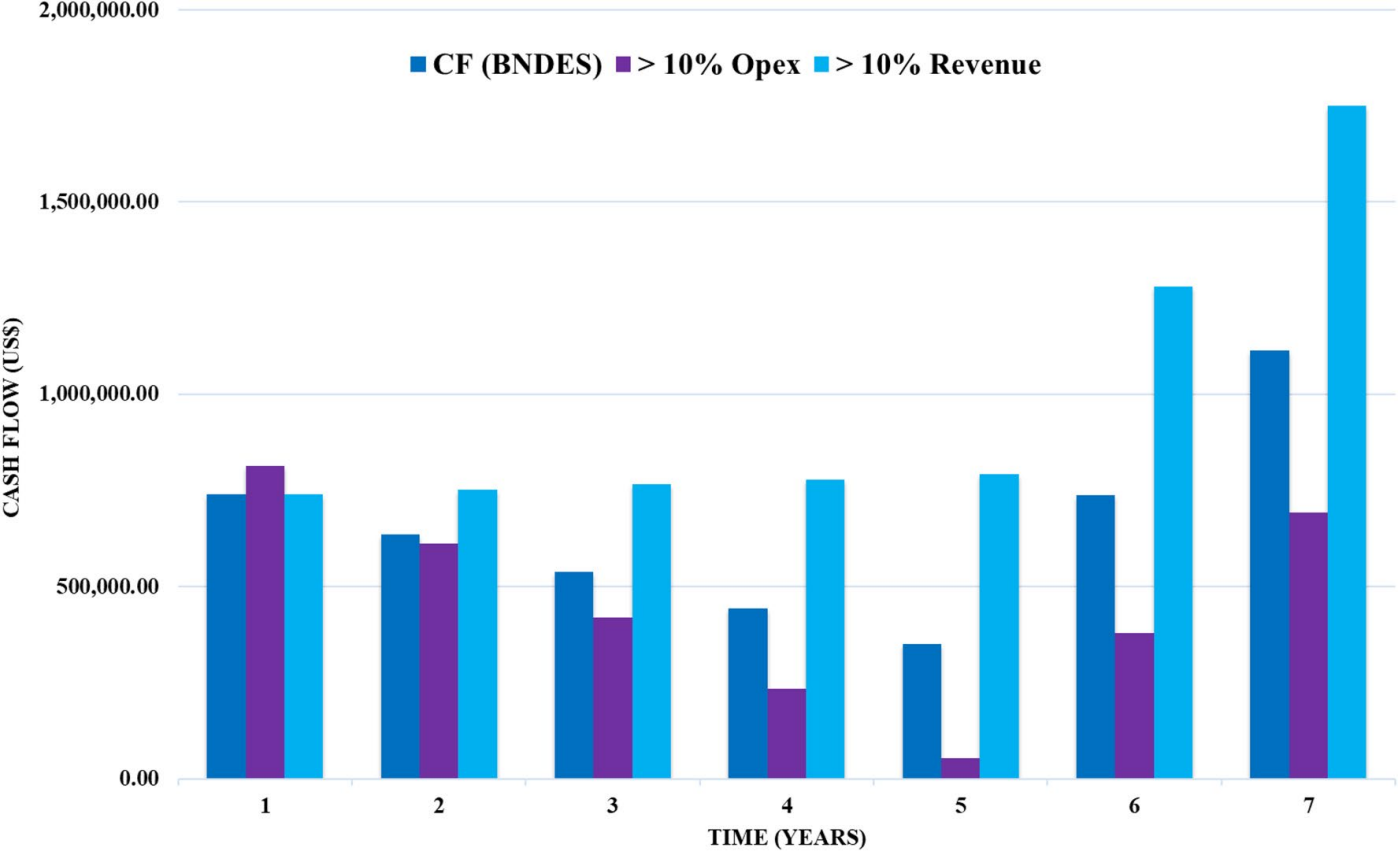
Cash flow with revenue evaluated and 10% increase in OPEX and revenue with bank loan.

The other option would be to seek a loan from a bank, in Brazil the BNDES lends amounts with interest at 12.4% per year. Using this option to cover CAPEX, 1 year of OPEX and returning the loan in 4 years, the results are also attractive, as can be seen in Fig. 8. The same 10% fluctuations in OPEX and revenue were considered.

## Conclusions

Based on the identified results, the developed biofuel can be characterized as a product rich in hydrocarbons. Further detailed analyses are necessary for process development. Expanding the temperature range and using other types of plastics, such as polypropylene and polyethylene, is also interesting since the waste contains a diverse range of polymers. The final proposal is to further reduce the sample size by using a ball mill with blades to evaluate the influence of surface area on the process. However, the technique has demonstrated efficiency in the synthesis of hydrocarbons from plastic and oily waste, providing an alternative route for waste management and alternative fuel synthesis.

In this work, a study of the use of plastic and oily residues for the synthesis of hydrogen, gasoline, ethylene and CH_4_ was proposed. The results of the simulation and economic evaluation point to a promising scenario. The total project investment was US$ 2,479,732.29 with an operating cost of US$ 739.654,83/year. The economic indicators reported the feasibility of the project under the conditions presented with payback time for all scenarios last than 5 years.

### Supplementary Information

Supplementary Tables.

## Data Availability

The datasets generated during and/or analyzed during the current study are available from the corresponding author on reasonable request.
