# Supplementary material for: Experimental study, simulation and technical–economic feasibility of an interesterification plant for hydrocarbons synthesis by using plastics and frying oil waste
Source: Sci Rep. 2024 May 3;14:10240. doi: 10.1038/s41598-024-60851-8 (PMC11068870; doi:10.1038/s41598-024-60851-8)
Supplement: Supplementary file 1 — Supplementary Tables. [file 41598_2024_60851_MOESM1_ESM.docx]

**SUPPLEMENTARY INFORMATION**

Experimental Study, Simulation and Technical-Economic Feasibility of An Interesterification Plant for Hydrocarbons Synthesis by Using Plastics and Frying Oil Waste

# Hugo Gomes D’Amato Villardi^1^+, Madson M. Nascimento^1,2,3^+, Fernando Luiz P. Pessoa^1,4^+, Alex Álisson B. Santos^1,4^+, Luiz Alberto Brêda Mascarenhas^1+^, Leone Peter Correia Andrade^1+^, Jailson B. de Andrade^1,2,3*^+

1Centro Universitário SENAI-CIMATEC, Av. Orlando Gomes, 1845 - Piatã, 41650-010, Salvador, BA, Brazil.

^2^Instituto Nacional de Ciência e Tecnologia em Energia e Ambiente – INCT E&A, Universidade Federal da Bahia, 40170-115 Salvador, BA, Brazil.

^3^Centro Interdisciplinar de Energia e Ambiente - CIEnAm, Universidade Federal da Bahia, 40170-115 Salvador, BA, Brazil ^4^Postgraduate Program in Computer Modeling and Industrial Technology.

*[jailsondeandrade@gmail.com](mailto:jailsondeandrade@gmail.com)

+these authors contributed equally to this work.

**Table contents**

**Table S1.** Chemical characterization of the volatile compounds profile in the reaction product obtained from of the test 1.1.1.

**Table S2.** Chemical characterization of the volatile compounds profile in the reaction product obtained from of the test 1.1.2.

**Table S3.** Chemical characterization of the volatile compounds profile in the reaction product obtained from of the test 1.1.3.

**Table S4.** Chemical characterization of the volatile compounds profile in the reaction product obtained from of the test 1.1.4.

**Table S5**. Chemical characterization of the volatile compounds profile in the reaction product obtained from of the test 1.1.5.

**Table S6.** Chemical characterization of the volatile compounds profile in the reaction product obtained from of the test 1.2.1.

**Table S7**. Chemical characterization of the volatile compounds profile in the reaction product obtained from of the test 1.2.2.

**Table S8.** Chemical characterization of the volatile compounds profile in the reaction product obtained from of the test 1.2.4.

**Table S9**. Chemical characterization of the volatile compounds profile in the reaction product obtained from of the test 1.2.5.

**Table S10**. Chemical characterization of the volatile compounds profile in the reaction product obtained from of the test 2.1.1.

**Table S11**. Chemical characterization of the volatile compounds profile in the reaction product obtained from of the test 2.1.2.

**Table S12.** Chemical characterization of the volatile compounds profile in the reaction product obtained from of the test 2.1.3.

**Table S13**. Chemical characterization of the volatile compounds profile in the reaction product obtained from of the test 2.1.4.

**Table S14**. Chemical characterization of the volatile compounds profile in the reaction product obtained from of the test 2.1.5.

**Table S16**. Chemical characterization of the volatile compounds profile in the reaction product obtained from of the test 2.2.1.

**Table 17**. Chemical characterization of the volatile compounds profile in the reaction product obtained from of the test 2.2.2.

**Table 18**. Chemical characterization of the volatile compounds profile in the reaction product obtained from of the test 2.2.3.

**Table S19**. Chemical characterization of the volatile compounds profile in the reaction product obtained from of the test 2.2.4.

**Table S20**. Chemical characterization of the volatile compounds profile in the reaction product obtained from of the test 2.2.5.

**Table S1.** Chemical characterization of the volatile compounds profile in the reaction product obtained from of the test 1.1.1. The detection of esters, short-chain hydrocarbons, and monoaromatics such as toluene as reaction products is observable.

|  |  |  |  |  |  |  |
| --- | --- | --- | --- | --- | --- | --- |
| **TR** | **Compound** | **Similarity index (%)** | **MW g/mol** | **Molecular formula** | **Area** | **Area (%)** |
| 2.672 | Ethyl acetate | 81 | 88 | C4H8O2 | 339070138 | 50.54 |
| 2.864 | 2-Methyl-1,3-dioxacyclopentane | 96 | 88 | C4H8O2 | 19595488 | 2.92 |
| 3.665 | 1-Propyl acetate | 91 | 102 | C5H10O2 | 795898 | 0.12 |
| 4.558 | Toluene | 95 | 92 | C7H8 | 182535 | 0.03 |
| 4.615 | 1-Methyl-1-cyclohexene | 93 | 96 | C7H12 | 172575 | 0.03 |
| 4.695 | 2-Methylpropyl acetate | 98 | 116 | C6H12O2 | 1589333 | 0.24 |
| 5.001 | 1-Octene | 96 | 112 | C8H16 | 7555433 | 1.13 |
| 5.283 | 2-Octene | 98 | 112 | C8H16 | 5218312 | 0.78 |
| 5.425 | 4-Octene | 94 | 112 | C8H16 | 2783632 | 0.41 |
| 5.542 | 1-Propylcyclopentene | 90 | 110 | C8H14 | 365574 | 0.05 |
| 5.763 | 1,2-dimethylcyclohexane | 91 | 112 | C8H16 | 108750 | 0.02 |
| 6.161 | Ethylene glycol monoacetate | 98 | 104 | C4H8O3 | 4309761 | 0.64 |
| 6.995 | 3-Nonene | 92 | 126 | C9H18 | 242073 | 0.04 |
| 7.114 | Acetato de ethoxyethyl | 98 | 132 | C6H12O3 | 1051276 | 0.16 |
| 8.696 | Ethylene glycol diacetate | 98 | 146 | C6H10O4 | 13366859 | 1.99 |
| 9.387 | D-limonene | 93 | 136 | C10H16 | 388783 | 0.06 |
| 9.494 | 1-pentyl-cyclopentane | 92 | 140 | C10H20 | 84028 | 0.01 |
| 10.308 | Ethyl heptanoate | 90 | 156 | C9H16O2 | 164168 | 0.02 |
| 10.394 | Octylcyclopropane | 93 | 168 | C12H24 | 134637 | 0.02 |
| 10.607 | 2-dodecene | 92 | 172 | C11H24O | 257263 | 0.04 |
| 10.750 | 2-undecene | 91 | 154 | C11H22 | 112145 | 0.02 |
| 10.984 | 3-Hexyl-1-cyclopentene | 93 | 152 | C11H20 | 532743 | 0.08 |
| 11.435 | 3-pentyl-cyclohexene | 92 | 152 | C11H20 | 102887 | 0.02 |
| 12.017 | Ethyl octanoate | 95 | 172 | C10H20O2 | 347876 | 0.05 |
|  | **Others*** |  |  |  | **272265217** | **40.58** |
|  | **Total** |  |  |  | **670923006** | **100.00** |

**Table S2.** Chemical characterization of the volatile compounds profile in the reaction product obtained from of the test 1.1.2. A notable amount of saturated hydrocarbons is observed, with minor detections of esters and cycloalkanes as well.

|  |  |  |  |  |  |  |
| --- | --- | --- | --- | --- | --- | --- |
| **TR** | **Compounds** | **Similarity index (%)** | **MW (g/mol)** | **Molecular formula** | **Area** | **Area(%)** |
| 2.510 | Ethyl acetate | 83 | 88 | C4H8O2 | 93383913 | 50.58 |
| 2.776 | 2-Methyldioxolane | 94 | 88 | C4H8O2 | 3545728 | 1.92 |
| 3.301 | 1-Heptene | 99 | 98 | C7H14 | 5526202 | 2.99 |
| 3.423 | Heptane | 95 | 100 | C7H16 | 554566 | 0.30 |
| 3.886 | 1.1-Diethoxyethane | 97 | 118 | C6H14O2 | 2661280 | 1.44 |
| 4.990 | 1-Octene | 94 | 112 | C8H16 | 1298917 | 0.70 |
| 5.269 | 2-Octene | 98 | 112 | C8H16 | 1333524 | 0.72 |
| 5.415 | 4-Octene | 96 | 112 | C8H16 | 661414 | 0.36 |
| 6.046 | Ethylene glycol monoacetate | 84 | 104 | C4H8O3 | 116109 | 0.06 |
| 6.993 | 3-Nonene | 91 | 126 | C9H18 | 28050 | 0.02 |
| 7.037 | 2,4-Dimethylhexane | 91 | 114 | C8H18 | 37098 | 0.02 |
| 7.108 | 2-Ethoxyethyl acetate | 96 | 132 | C6H12O3 | 78471 | 0.04 |
| 8.170 | Dicyclopropylmethane | 91 | 96 | C7H12 | 4987 | 0.003 |
| 9.889 | 3-Butenylcyclopropane | 92 | 96 | C7H12 | 1012 | 0.00 |
| 10.397 | 3,5-Dimethyl-1-hexene | 90 | 112 | C8H16 | 13037 | 0.01 |
| 10.535 | 1,2-Dineopentyldisulfane | 96 | 254 | C10H22O3S2 | 2855 | 0.00 |
| 10.610 | 3,5-Dimethyl-1-hexene | 93 | 112 | C8H16 | 25745 | 0.01 |
|  | **Others*** | **-** | **-** | **-** | **75354855** | **40.81** |
|  | **Total** | **-** | **-** | **-** | **184635506** | **100.00** |

*Including unidentified compounds and column bleeding products.

Table S3. Chemical characterization of the volatile compounds profile in the reaction product obtained from of the test 1.1.3. A significant quantity of saturated hydrocarbons is observable, including some esters and monoaromatics.

|  | **1.1.3** |  |  |  |  |  |
| --- | --- | --- | --- | --- | --- | --- |
| **TR** | **Compound** | **Similarity index (%)** | **MW g/mol** | **Molecular formula** | **Area** | **Area (%)** |
| 2.225 | Propylcyclopropane | 94 | 84 | C6H12 | 121863 | 0.120 |
| 2.286 | 3-Methyl pentanecetate | 94 | 86 | C6H14 | 506901 | 0.501 |
| 2.458 | Ethyl acetate | 83 | 88 | C4H8O2 | 53251669 | 52.623 |
| 2.786 | Methyldioxolane | 94 | 88 | C4H8O2 | 442796 | 0.438 |
| 3.307 | 1-Heptene | 98 | 98 | C7H14 | 2800781 | 2.768 |
| 3.431 | Heptane | 95 | 100 | C7H16 | 252485 | 0.250 |
| 3.540 | 2-Heptene | 95 | 98 | C7H14 | 206171 | 0.204 |
| 3.902 | Diethyl acetal | 98 | 118 | C6H14O2 | 967984 | 0.957 |
| 4.549 | Toluene | 95 | 92 | C7H8 | 24382 | 0.024 |
| 4.992 | 1-Octene | 96 | 112 | C8H16 | 646541 | 0.639 |
| 5.166 | Octane | 89 | 114 | C8H18 | 399852 | 0.395 |
| 5.272 | 4-Octene | 96 | 112 | C8H16 | 764027 | 0.755 |
| 5.418 | 3-Octene | 95 | 112 | C8H16 | 410067 | 0.405 |
| 6.989 | 3-Nonene | 90 | 126 | C9H18 | 15936 | 0.016 |
| 7.036 | 2,4-Dimethylhexane | 90 | 114 | C8H18 | 17191 | 0.017 |
| 7.280 | Cyclobutanone | 91 | 112 | C7H12O | 6923 | 0.007 |
|  | **Others** |  |  |  | **40358414** | **39.882** |
|  | **Total** |  |  |  | **101193983** | **100.000** |

Table S4. Chemical characterization of the volatile compounds profile in the reaction product obtained from of the test 1.1.4. A significant quantity of saturated hydrocarbons was detected.

|  | **1.1.4** |  |  |  |  |  |
| --- | --- | --- | --- | --- | --- | --- |
| **TR** | **Compound** | **Similarity index (%)** | **MW g/mol** | **Molecular formula** | **Area** | **Area (%)** |
| 2.544 | Ethyl acetate | 83 | 88 | C4H8O2 | 104746637 | 41.042 |
| 2.797 | 2-Methyldioxolane | 92 | 88 | C4H8O2 | 4154996 | 1.628 |
| 3.899 | Ethylidene diethyl ether | 97 | 118 | C6H14O2 | 2927312 | 1.147 |
| 4.553 | Toluene | 91 | 92 | C7H8 | 391031 | 0.153 |
| 5.001 | 1-Octene | 96 | 112 | C8H16 | 3571640 | 1.399 |
| 5.282 | 2-Octene | 98 | 112 | C8H16 | 4721728 | 1.850 |
| 5.425 | 4-Octene | 94 | 112 | C8H16 | 2575846 | 1.009 |
| 6.007 | Ethyl 2-butenoate | 93 | 114 | C6H10O2 | 202328 | 0.079 |
| 6.966 | trans-4-Nonene | 94 | 126 | C9H18 | 364936 | 0.143 |
| 6.996 | 4-Nonene | 92 | 126 | C9H18 | 205770 | 0.081 |
| 7.041 | Nonane | 92 | 128 | C9H20 | 450005 | 0.176 |
| 7.116 | Ethyl glycol acetate | 95 | 132 | C6H12O3 | 78251 | 0.031 |
| 7.144 | 2-Nonene | 95 | 126 | C9H18 | 254080 | 0.100 |
| 7.286 | cis-2-Nonene | 93 | 126 | C9H18 | 145090 | 0.057 |
| 7.437 | 3-Butyl-1-cyclopentene | 92 | 124 | C9H16 | 46577 | 0.018 |
| 7.953 | 1-Propyl-1-cyclohexene | 91 | 124 | C9H16 | 90175 | 0.035 |
| 8.261 | 3-Ethyloctane | 93 | 142 | C10H22 | 29765 | 0.012 |
| 8.736 | cis-5-Decene | 97 | 140 | C10H20 | 339657 | 0.133 |
| 9.081 | 1-Heptene | 91 | 98 | C7H14 | 17142 | 0.007 |
| 9.265 | 3-Pentyl-1-cyclopentene | 90 | 138 | C10H18 | 97830 | 0.038 |
| 9.498 | Pentylcyclopentane | 92 | 140 | C10H20 | 56994 | 0.022 |
| 9.750 | 3-Butyl-1-cyclohexene | 90 | 138 | C10H18 | 93065 | 0.036 |
| 10.400 | 3.5-Dimethyl-1-hexene | 91 | 112 | C8H16 | 54412 | 0.021 |
| 10.611 | 2-Undecene | 91 | 154 | C11H22 | 55337 | 0.022 |
| 10.986 | 3-Hexyl-1-cyclopentene | 90 | 110 | C8H14 | 124619 | 0.049 |
| 11.440 | 3-Pentyl-1-cyclohexene | 94 | 152 | C11H20 | 82056 | 0.032 |
| 11.486 | Pentylbenzene | 92 | 148 | C11H16 | 11957 | 0.005 |
|  | Others |  |  |  | 129327961 | 50.674 |
|  | Total |  |  |  | 255217197 | 100.000 |

Table S5. Chemical characterization of the volatile compounds profile in the reaction product obtained from of the test 1.1.5. A significant quantity of saturated hydrocarbons was detected. Some alcohols and esters were detected as minority compounds.

|  | **1.1.5** |  |  |  |  |  |
| --- | --- | --- | --- | --- | --- | --- |
| **TR** | **Compound** | **Similarity index (%)** | **MW g/mol** | **Molecular formula** | **Area** | **Area (%)** |
| 2.223 | 1-Hexanol | 95 | 84 | C6H12 | 5252621 | 1.486 |
| 2.286 | 3-Methyl pentane | 94 | 86 | C6H14 | 9426587 | 2.667 |
| 2.569 | Ethyl Acetate | 84 | 88 | C4H8O2 | 141491375 | 40.032 |
| 2.808 | Methyldioxolane | 96 | 88 | C4H8O2 | 7045122 | 1.993 |
| 3.174 | Ethenylcyclobutane | 94 | 82 | C6H10 | 9300169 | 2.631 |
| 3.319 | 1-Heptene | 98 | 98 | C7H14 | 24614521 | 6.964 |
| 3.440 | Heptane | 96 | 100 | C7H16 | 8475636 | 2.398 |
| 3.556 | 2-Heptene | 95 | 98 | C7H14 | 10283295 | 2.909 |
| 3.906 | Ethylidene diethyl ether | 96 | 118 | C6H14O2 | 4553562 | 1.288 |
| 4.105 | 3-Methylcyclohexene | 94 | 96 | C7H12 | 2131272 | 0.603 |
| 4.555 | Toluene | 97 | 92 | C7H8 | 847528 | 0.240 |
| 5.003 | 1-Octene | 96 | 112 | C8H16 | 5681860 | 1.608 |
| 5.285 | 2-Octene | 98 | 112 | C8H16 | 7794007 | 2.205 |
| 5.428 | trans-2-Octene | 95 | 112 | C8H16 | 4636773 | 1.312 |
| 6.011 | Ethyl 2-butenoate | 95 | 114 | C6H10O2 | 520750 | 0.147 |
| 7.001 | 4-Nonene | 93 | 126 | C9H18 | 1645964 | 0.466 |
| 7.048 | Nonane | 91 | 128 | C9H20 | 973008 | 0.275 |
| 7.146 | 2-Ethoxyethyl acetate | 98 | 132 | C6H12O3 | 1109129 | 0.314 |
| 7.212 | cis-2-Nonene | 92 | 126 | C9H18 | 237160 | 0.067 |
| 7.288 | 3-Nonene | 94 | 126 | C9H18 | 297212 | 0.084 |
| 7.956 | 1-Propyl-1-cyclohexene | 92 | 124 | C9H16 | 378134 | 0.107 |
| 8.261 | 2.3-Dimethylnonane | 91 | 156 | C11H24 | 174423 | 0.049 |
| 8.336 | 3-Methylnonane | 92 | 142 | C10H22 | 89899 | 0.025 |
| 8.737 | (Z)-5-Decene | 97 | 140 | C10H20 | 1413415 | 0.400 |
| 8.850 | Decane | 90 | 142 | C10H22 | 209254 | 0.059 |
| 9.499 | Pentylcyclopentane | 94 | 140 | C10H20 | 199656 | 0.056 |
| 9.750 | 3-Butyl-1-cyclohexene | 93 | 138 | C10H18 | 218988 | 0.062 |
| 10.401 | Octylcyclopropane | 93 | 154 | C11H22 | 250961 | 0.071 |
| 10.462 | Ethyl heptanoate | 93 | 158 | C9H18O2 | 216180 | 0.061 |
| 10.613 | 2-Undecene | 92 | 154 | C11H22 | 448476 | 0.127 |
| 10.754 | 1-Heptyl-2-methylcyclopropane | 92 | 154 | C11H22 | 240243 | 0.068 |
| 10.985 | 3-Isopentyl-1-cyclopentene | 92 | 152 | C11H20 | 492718 | 0.139 |
| 11.441 | 3-Pentyl-1-cyclohexene | 96 | 152 | C11H20 | 335972 | 0.095 |
| 13.027 | 3-Hexyl-1-cyclohexene | 90 | 152 | C11H20 | 92906 | 0.026 |
|  | Others |  |  |  | 102366464 | 28.962 |
|  | Total |  |  |  | 353445240 | 100.000 |

Table S6. Chemical characterization of the volatile compounds profile in the reaction product obtained from of the test 1.2.1. A significant quantity of saturated hydrocarbons was detected. Some alcohols and esters were detected as minority compounds.

|  | **1.2.1** |  |  |  |  |  |
| --- | --- | --- | --- | --- | --- | --- |
| **TR** | **Compound** | **Similarity index (%)** | **MW g/mol** | **Molecular formula** | **Area** | **Area (%)** |
| 2.074 | Ethanol | 99 | 46 | C2H6O | 17882534 | 8.256 |
| 2.573 | Ethyl Acetate | 84 | 88 | C4H8O2 | 175270017 | 80.915 |
| 2.846 | 1,3-Dioxolane | 93 | 88 | C4H8O2 | 7533054 | 3.478 |
| 3.273 | 1-Heptene | 95 | 98 | C7H14 | 361875 | 0.167 |
| 3.505 | 2-Heptene | 91 | 98 | C7H14 | 197441 | 0.091 |
| 4.038 | 1,1-Diethoxyethane | 98 | 118 | C6H14O2 | 3386244 | 1.563 |
| 4.378 | 2-Ethyl-1.3-dioxolane | 94 | 102 | C5H10O2 | 63548 | 0.029 |
| 4.757 | 2-Methylpropyl acetate | 93 | 116 | C6H12O2 | 186542 | 0.086 |
| 5.204 | Hexanal | 92 | 100 | C6H12O | 766205 | 0.354 |
| 5.261 | 2-Octene | 96 | 112 | C8H16 | 708148 | 0.327 |
| 6.993 | 3-Nonene | 90 | 126 | C9H18 | 109698 | 0.051 |
| 7.283 | 2.3.3-Trimethylcyclobutanone | 92 | 112 | C7H12O | 13267 | 0.006 |
| 9.266 | 1.1'-Bicyclobutyl | 92 | 110 | C7H10O | 55419 | 0.026 |
| 10.399 | 3-Methyl-1-hexanol | 90 | 98 | C7H14 | 21414 | 0.010 |
| 10.535 | Undecane | 92 | 156 | C11H24 | 52284 | 0.024 |
| 10.611 | 3.5-Dimethyl-1-hexene | 94 | 112 | C8H16 | 35570 | 0.016 |
| 11.482 | Toluene | 98 | 92 | C7H8 | 4430 | 0.002 |
|  | Others |  |  |  | 9961171 | 4.599 |
|  | Total |  |  |  | 216608861 | 100.000 |

Table S7. Chemical characterization of the volatile compounds profile in the reaction product obtained from of the test 1.2.2. A significant quantity of saturated hydrocarbons was detected.

|  | **1.2.2** |  |  |  |  |  |
| --- | --- | --- | --- | --- | --- | --- |
| **TR** | **Compound** | **Similarity index (%)** | **MW g/mol** | **Molecular formula** | **Area** | **Area (%)** |
| 1.651 | Oxoacetic acid | 97 | 74 | C2H2O3 | 5121676 | 2.043 |
| 2.014 | 1-Cyclopentene | 92 | 68 | C5H8 | 249953 | 0.100 |
| 2.226 | 1-Hexene | 95 | 84 | C6H12 | 1570585 | 0.626 |
| 2.289 | 3-Methyl pentane | 94 | 86 | C6H14 | 4690412 | 1.871 |
| 2.558 | Ethyl Acetate | 84 | 88 | C4H8O2 | 128535806 | 51.264 |
| 2.804 | Methyldioxolane | 92 | 88 | C4H8O2 | 13133128 | 5.238 |
| 3.318 | 1-Heptene | 98 | 98 | C7H14 | 11983627 | 4.779 |
| 3.439 | 1-Heptene | 96 | 100 | C7H16 | 2372429 | 0.946 |
| 3.560 | 2-Heptene | 91 | 98 | C7H14 | 2452208 | 0.978 |
| 3.788 | 2-Norbornene | 94 | 94 | C7H10 | 1447334 | 0.577 |
| 3.906 | Ethylidene diethyl ether | 97 | 118 | C6H14O2 | 4109514 | 1.639 |
| 4.190 | Tricyclo[2,2,1,0(2,6)]heptane | 91 | 94 | C7H10 | 258800 | 0.103 |
| 4.495 | Alpha-Benzenemethanol | 91 | 58 | C3H6O | 62781 | 0.025 |
| 4.556 | Toluene | 91 | 92 | C7H8 | 261877 | 0.104 |
| 4.617 | 1-Methyl-1-cyclohexene | 95 | 96 | C7H12 | 456822 | 0.182 |
| 5.004 | 1-Octene | 97 | 112 | C8H16 | 3400721 | 1.356 |
| 5.284 | trans-2-Octene | 98 | 112 | C8H16 | 3880322 | 1.548 |
| 5.428 | cis-2-Octene | 96 | 112 | C8H16 | 2343932 | 0.935 |
| 5.771 | 1.2-Dimethylcyclohexane | 92 | 112 | C8H16 | 87998 | 0.035 |
| 6.008 | Ethyl 2-butenoate | 90 | 114 | C6H10O2 | 144507 | 0.058 |
| 6.997 | trans-3-Nonene | 95 | 126 | C9H18 | 606090 | 0.242 |
| 7.113 | 2-Ethoxyethyl acetate | 97 | 132 | C6H12O3 | 283095 | 0.113 |
| 7.144 | 2-Nonene | 94 | 126 | C9H18 | 219179 | 0.087 |
| 7.285 | cis-2-Nonene | 91 | 126 | C9H18 | 131133 | 0.052 |
| 8.737 | cis-5-Decene | 96 | 140 | C10H20 | 606463 | 0.242 |
| 9.265 | 3-Pentyl-1-cyclopentene | 92 | 138 | C10H18 | 274509 | 0.109 |
| 9.390 | alpha.-Limonene | 91 | 136 | C10H16 | 114247 | 0.046 |
| 9.498 | Pentylcyclopentane | 92 | 140 | C10H20 | 93647 | 0.037 |
| 10.310 | Ethyl 6-heptenoate | 91 | 156 | C9H16O2 | 184654 | 0.074 |
| 10.400 | Octylcyclopropane | 94 | 154 | C11H22 | 156879 | 0.063 |
| 10.462 | Ethyl heptanoate | 94 | 158 | C9H18O2 | 171105 | 0.068 |
| 10.612 | cis-2-Undecene | 95 | 154 | C11H22 | 333392 | 0.133 |
| 10.754 | cis-3-Undecene | 93 | 154 | C11H22 | 134516 | 0.054 |
| 11.490 | 3-Pentyl-1-cyclohexene | 91 | 148 | C11H16 | 89075 | 0.036 |
|  | Others |  |  |  | 60770874 | 24.237 |
|  | Total |  |  |  | 250733290 | 100.000 |

Table S8. Chemical characterization of the volatile compounds profile in the reaction product obtained from of the test 1.2.4. A significant quantity of saturated hydrocarbons was detected as well as some aromatics such as benzene and toluene.

|  | **1.2.4** |  |  |  |  |  |
| --- | --- | --- | --- | --- | --- | --- |
| **TR** | **Compound** | **Similarity index (%)** | **MW g/mol** | **Molecular formula** | **Area** | **Area (%)** |
| 2.168 | 2,2,3-Trimethylhexane | 91 | 128 | C9H20 | 148122 | 0.049 |
| 2.288 | Hexane | 95 | 86 | C6H14 | 6793592 | 2.260 |
| 2.539 | Ethyl acetate | 83 | 88 | C4H8O2 | 130846943 | 43.536 |
| 2.802 | 2-Methyldioxolane | 97 | 88 | C4H8O2 | 11178605 | 3.719 |
| 2.930 | Benzene | 90 | 78 | C6H6 | 2338920 | 0.778 |
| 3.172 | Cyclobutane | 95 | 82 | C6H10 | 6045393 | 2.011 |
| 3.317 | 1-Heptene | 99 | 98 | C7H14 | 22253748 | 7.404 |
| 3.436 | Heptane | 93 | 100 | C7H16 | 5596485 | 1.862 |
| 3.551 | trans-2-Heptene | 97 | 98 | C7H14 | 5676425 | 1.889 |
| 3.905 | Ethylidene diethyl ether | 98 | 118 | C6H14O2 | 2550099 | 0.848 |
| 4.090 | 3-Methylcyclohexene | 95 | 96 | C7H12 | 1383463 | 0.460 |
| 4.554 | Toluene | 97 | 92 | C7H8 | 595473 | 0.198 |
| 4.615 | 1-Methylcyclohexene | 97 | 96 | C7H12 | 685773 | 0.228 |
| 4.999 | 1-Octene | 97 | 112 | C8H16 | 4468753 | 1.487 |
| 5.281 | trans-2-Octene | 98 | 112 | C8H16 | 5845556 | 1.945 |
| 5.425 | 2-Octene | 96 | 112 | C8H16 | 3288981 | 1.094 |
| 5.542 | 3-Propylcyclopentene | 92 | 110 | C8H14 | 366686 | 0.122 |
| 5.773 | 1,2-Dimethylcyclohexane | 93 | 112 | C8H16 | 180356 | 0.060 |
| 6.520 | 3-Methyloctane | 92 | 128 | C9H20 | 200113 | 0.067 |
| 6.885 | 1-Nonene | 95 | 126 | C9H18 | 576216 | 0.192 |
| 6.966 | trans--4-Nonene | 94 | 126 | C9H18 | 407180 | 0.135 |
| 6.995 | trans-3-Nonene | 95 | 126 | C9H18 | 560448 | 0.186 |
| 7.044 | Nonane | 91 | 128 | C9H20 | 557007 | 0.185 |
| 7.126 | 2-Ethoxyethyl acetate | 98 | 132 | C6H12O3 | 1672610 | 0.557 |
| 7.441 | 3-Butyl-1-cyclopentene | 94 | 124 | C9H16 | 65785 | 0.022 |
| 7.956 | 1-Propyl-1-cyclohexene | 91 | 124 | C9H16 | 213396 | 0.071 |
| 8.265 | 3-Ethyloctane | 92 | 142 | C10H22 | 86726 | 0.029 |
| 8.336 | 2,2-Dimethylbutane | 90 | 86 | C6H14 | 40808 | 0.014 |
| 8.738 | cis-4-Decene | 97 | 140 | C10H20 | 998504 | 0.332 |
| 9.500 | Pentylcyclopentane | 93 | 140 | C10H20 | 76497 | 0.025 |
| 10.312 | Ethyl 6-heptenoate | 92 | 156 | C9H16O2 | 88694 | 0.030 |
| 10.464 | Octylcyclopropane | 94 | 154 | C11H22 | 112853 | 0.038 |
| 10.612 | 3-Undecene | 93 | 154 | C11H22 | 170471 | 0.057 |
| 10.987 | 3-Hexyl-1-cyclopentene | 91 | 152 | C11H20 | 257919 | 0.086 |
| 11.441 | 3-Pentyl-1-cyclohexene | 95 | 152 | C11H20 | 141933 | 0.047 |
|  | Others |  |  |  | 84078448 | 27.975 |
|  | Total |  |  |  | 300548981 | 100.000 |

Table S9. Chemical characterization of the volatile compounds profile in the reaction product obtained from of the test 1.2.5. A significant quantity of saturated hydrocarbons was detected as well as some esters, and aromatics such as benzene and toluene.

|  | **1.2.5** |  |  |  |  |  |
| --- | --- | --- | --- | --- | --- | --- |
| **TR** | **Compound** | **Similarity index (%)** | **MW g/mol** | **Molecular formula** | **Area** | **Area (%)** |
| 1.634 | Glyoxylic acidate | 95 | 74 | C2H2O3 | 3551058 | 1.029 |
| 2.161 | 3-Methylpentane | 95 | 86 | C6H14 | 153571 | 0.045 |
| 2.219 | Propylcyclopropane | 94 | 84 | C6H12 | 3221383 | 0.934 |
| 2.281 | 3-Methyl pentane | 95 | 86 | C6H14 | 5783494 | 1.676 |
| 2.528 | Ethyl acetate | 83 | 88 | C4H8O2 | 115779926 | 33.558 |
| 2.801 | Methyldioxolane | 95 | 88 | C4H8O2 | 8388835 | 2.431 |
| 2.928 | Benzene | 97 | 78 | C6H6 | 5696049 | 1.651 |
| 3.171 | Cyclohexene | 97 | 82 | C6H10 | 10176136 | 2.949 |
| 3.316 | 1-Heptene | 96 | 98 | C7H14 | 25701562 | 7.449 |
| 3.839 | 1-Cyano-2-propenyl acetate | 91 | 125 | C6H7NO2 | 2080723 | 0.603 |
| 4.101 | 3-Methylcyclohexene | 90 | 96 | C7H12 | 2812611 | 0.815 |
| 4.553 | Toluene | 96 | 92 | C7H8 | 1472115 | 0.427 |
| 4.614 | 1-Methylcyclohexene | 93 | 96 | C7H12 | 1397381 | 0.405 |
| 4.658 | 3-Methylheptane | 94 | 114 | C8H18 | 1984736 | 0.575 |
| 5.002 | 1-Octene | 96 | 112 | C8H16 | 8180494 | 2.371 |
| 5.285 | trans-2-Octene | 97 | 112 | C8H16 | 11080850 | 3.212 |
| 5.428 | cis-2-Octene | 93 | 112 | C8H16 | 6651906 | 1.928 |
| 5.542 | 3-Propylcyclopentene | 92 | 110 | C8H14 | 691183 | 0.200 |
| 5.960 | 1-Cyclopropyl-1-dodecanone | 90 | 224 | C15H28O | 200737 | 0.058 |
| 6.885 | 1-Nonene | 96 | 126 | C9H18 | 1819057 | 0.527 |
| 6.965 | trans--4-Nonene | 97 | 126 | C9H18 | 1492638 | 0.433 |
| 6.997 | cis-2-Nonene | 95 | 126 | C9H18 | 1493616 | 0.433 |
| 7.044 | Nonane | 92 | 128 | C9H20 | 1696790 | 0.492 |
| 7.140 | cis-2-Nonene | 90 | 126 | C9H18 | 2521820 | 0.731 |
| 7.290 | 2-Nonene | 93 | 126 | C9H18 | 641343 | 0.186 |
| 7.441 | 3-Isobutyl-1-cyclopentene | 94 | 124 | C9H16 | 246800 | 0.072 |
| 7.580 | Ethyl 2-ethylbutanoate | 90 | 144 | C8H16O2 | 288100 | 0.084 |
| 7.958 | 1-Propyl-1-cyclohexene | 93 | 124 | C9H16 | 696182 | 0.202 |
| 8.264 | 2,3-Dimethylnonane | 94 | 156 | C11H24 | 245648 | 0.071 |
| 8.337 | 3-Methylnonane | 95 | 142 | C10H22 | 230973 | 0.067 |
| 8.739 | cis-5-Decene | 96 | 140 | C10H20 | 3333060 | 0.966 |
| 9.268 | 3-Pentyl-1-cyclopentene | 93 | 138 | C10H18 | 718282 | 0.208 |
| 9.501 | Pentylcyclopentane | 92 | 140 | C10H20 | 499948 | 0.145 |
| 9.754 | 3-Butyl-1-cyclohexene | 92 | 138 | C10H18 | 681096 | 0.197 |
| 9.840 | Butylbenzene | 91 | 134 | C10H14 | 141532 | 0.041 |
| 10.314 | Ethyl 6-heptenoate | 92 | 156 | C9H16O2 | 329226 | 0.095 |
| 10.405 | Octylcyclopropane | 97 | 154 | C11H22 | 549353 | 0.159 |
| 10.466 | Ethyl heptanoate | 93 | 158 | C9H18O2 | 467707 | 0.136 |
| 10.615 | cis-2-Undecene | 94 | 154 | C11H22 | 665795 | 0.193 |
| 10.757 | cis-3-Undecene | 93 | 154 | C11H22 | 517765 | 0.150 |
| 10.988 | 3-Hexyl-1-cyclopentene | 93 | 152 | C11H20 | 852252 | 0.247 |
| 11.444 | 3-Pentyl-1-cyclohexene | 98 | 152 | C11H20 | 712881 | 0.207 |
| 11.488 | n-Pentyl benzene | 92 | 148 | C11H16 | 286449 | 0.083 |
| 11.690 | Ethyl benzoate | 94 | 150 | C9H10O2 | 80339 | 0.023 |
| 11.950 | (6E)-6-Dodecene | 96 | 168 | C12H24 | 225468 | 0.065 |
| 11.989 | (4Z)-4-Dodecene | 95 | 168 | C12H24 | 201200 | 0.058 |
| 12.023 | Ethyl octanoate | 94 | 172 | C10H20O2 | 416524 | 0.121 |
| 13.030 | 3-Hexyl-1-cyclohexene | 90 | 166 | C12H22 | 261498 | 0.076 |
| 13.414 | (7Z)-7-Tetradecene | 92 | 196 | C14H28 | 64396 | 0.019 |
|  | **Others** |  |  |  | **107633301** | **31.197** |
|  | **Total** |  |  |  | **345015789** | **100.000** |

Table S10. Chemical characterization of the volatile compounds profile in the reaction product obtained from of the test 2.1.1. A reduced quantity of compounds were detected. Esters were the major compounds in this sample.

|  | **2.1.1** |  |  |  |  |  |
| --- | --- | --- | --- | --- | --- | --- |
| **TR** | **Compound** | **Similarity index (%)** | **MW g/mol** | **Molecular formula** | **Area** | **Area (%)** |
| 2.983 | Ethyl Acetate | 82 | 88 | C4H8O2 | 657623665 | 97.596 |
| 3.767 | Ethyl propanoate | 99 | 102 | C5H10O2 | 1731525 | 0.257 |
| 3.805 | Propyl acetate | 94 | 102 | C5H10O2 | 1683233 | 0.250 |
| 4.191 | Isopropylacetone | 95 | 100 | C6H12O | 81356 | 0.012 |
| 4.373 | Ethylene glycol, formal | 97 | 74 | C3H6O2 | 11505 | 0.002 |
| 4.643 | Toluene | 99 | 92 | C7H8 | 14182 | 0.002 |
| 4.771 | 2-Methylpropyl acetate | 96 | 116 | C6H12O2 | 382883 | 0.057 |
| 7.755 | 2,2-Ethylenedioxy-6-methyl-5-methylideneheptane | 95 | 184 | C11H20O2 | 1435 | 0.000 |
|  | Others |  |  |  | 12291326 | 1.824 |
|  | Total |  |  |  | 673821110 | 100.000 |

Table S11. Chemical characterization of the volatile compounds profile in the reaction product obtained from of the test 2.1.2. A reduced quantity of compounds were detected. Esters were the major compounds in this sample.

|  | **2.1.2** |  |  |  |  |  |
| --- | --- | --- | --- | --- | --- | --- |
| **TR** | **Compound** | **Similarity index (%)** | **MW g/mol** | **Molecular formula** | **Area** | **Area (%)** |
| 1.640 | Ethanol | 98 | 46 | C2H6O | 689687 | 0.246 |
| 2.660 | Ethyl acetate | 82 | 88 | C4H8O2 | 237570304 | 84.907 |
| 3.643 | Ethyl propanoate | 98 | 102 | C5H10O2 | 533804 | 0.191 |
| 3.965 | Acetic acid | 92 | 60 | C2H4O2 | 258243 | 0.092 |
| 4.094 | 2-Methylpropyl methyl ketone | 94 | 100 | C6H12O | 192700 | 0.069 |
| 4.556 | Toluene | 98 | 92 | C7H8 | 19364 | 0.007 |
| 4.699 | 2-Methyl-1-propyl acetate | 96 | 116 | C6H12O2 | 967822 | 0.346 |
| 6.317 | Ethyl benzene | 91 | 106 | C8H10 | 131620 | 0.047 |
| 6.612 | Butenone | 96 | 70 | C4H6O | 19077 | 0.007 |
| 6.893 | Styrene | 98 | 104 | C8H8 | 649891 | 0.232 |
| 7.333 | Ethylene glycol, formal | 94 | 74 | C3H6O2 | 5252 | 0.002 |
| 9.813 | Ethylene glycol, formal | 91 | 172 | C10H20O2 | 35883 | 0.013 |
|  | **Others** |  |  |  | **38725975** | **13.841** |
|  | **Total** |  |  |  | **279799622** | **100.000** |

Table S12. Chemical characterization of the volatile compounds profile in the reaction product obtained from of the test 2.1.3. This sample was characterized by the high number of monoaromatic such as toluene, xylene, and styrene.

|  | **2.1.3** |  |  |  |  |  |
| --- | --- | --- | --- | --- | --- | --- |
| **TR** | **Compound** | **Similarity index (%)** | **MW g/mol** | **Molecular formula** | **Area** | **Area (%)** |
| 1.665 | Ethanol | 98 | 46 | C2H6O | 16290764 | 2.057 |
| 1.967 | Formic acid | 96 | 46 | CH2O2 | 26494187 | 3.345 |
| 3.005 | Ethyl acetate | 82 | 88 | C4H8O2 | 696705094 | 87.959 |
| 3.376 | 2,3-Dihydro-1.4-dioxine | 96 | 86 | C4H6O2 | 45082 | 0.006 |
| 3.727 | 1,4-Dioxacyclohexane | 97 | 88 | C4H8O2 | 141637 | 0.018 |
| 3.778 | Ethyl propanoate | 99 | 102 | C5H10O2 | 2401258 | 0.303 |
| 3.816 | Propyl acetate | 94 | 102 | C5H10O2 | 2394219 | 0.302 |
| 4.031 | 1,1-Diethoxyethane | 93 | 118 | C6H14O2 | 34755 | 0.004 |
| 4.197 | 2-Methylpropyl methyl ketone | 97 | 100 | C6H12O | 177527 | 0.022 |
| 4.376 | 2-Ethyl-1.3-dioxolane | 97 | 102 | C5H10O2 | 226335 | 0.029 |
| 4.465 | 2-Methylimidazoline | 93 | 84 | C4H8N2 | 14191 | 0.002 |
| 4.652 | Toluene | 95 | 92 | C7H8 | 51898 | 0.007 |
| 4.776 | 2-Methylpropyl acetate | 97 | 116 | C6H12O2 | 729506 | 0.092 |
| 5.070 | 1-Octene | 92 | 112 | C8H16 | 114164 | 0.014 |
| 5.237 | Hexanal | 92 | 100 | C6H12O | 455664 | 0.058 |
| 6.351 | Ethylbenzene | 95 | 106 | C8H10 | 86214 | 0.011 |
| 6.518 | p-Xylene | 95 | 106 | C8H10 | 16331 | 0.002 |
| 6.916 | Styrene | 95 | 104 | C8H8 | 120329 | 0.015 |
| 7.498 | Acetophenone | 96 | 120 | C8H8O | 6449 | 0.001 |
| 9.230 | 3-Methylhexane | 93 | 100 | C7H16 | 2438 | 0.000 |
| 9.329 | 4,4-Dimethyl-1-pentene | 90 | 98 | C7H14 | 26503 | 0.003 |
| 9.880 | 1,2-Dineopentyldisulfane 1,1,2-trioxide | 98 | 254 | C10H22O3S2 | 1379 | 0.000 |
|  | Others |  |  |  | 45547443 | 5.750 |
|  | Total |  |  |  | 792083367 | 100.000 |

Table S13. Chemical characterization of the volatile compounds profile in the reaction product obtained from of the test 2.1.4. This sample was characterized by the high number of monoaromatic such as toluene, xylene, ethylbenzene, and styrene.

|  | **2.1.4** |  |  |  |  |  |
| --- | --- | --- | --- | --- | --- | --- |
| **TR** | **Compound** | **Similarity index (%)** | **MW g/mol** | **Molecular formula** | **Area** | **Area (%)** |
| 1.652 | Ethanol | 98 | 46 | C2H6O | 7900762 | 1.754 |
| 1.803 | 2,2-Dihydroxymalonic acid | 93 | 136 | C3H4O6 | 18039860 | 4.006 |
| 2.788 | Ethyl acetate | 82 | 88 | C4H8O2 | 386975911 | 85.923 |
| 3.246 | 2,3-Dihydro-1.4-dioxine | 95 | 86 | C4H6O2 | 91999 | 0.020 |
| 3.383 | 1-heptene | 92 | 98 | C7H14 | 119592 | 0.027 |
| 3.635 | 1,4-Diethylene dioxide | 96 | 88 | C4H8O2 | 46435 | 0.010 |
| 3.687 | Ethyl propanoate | 99 | 102 | C5H10O2 | 2640096 | 0.586 |
| 3.729 | Propyl acetate | 94 | 102 | C5H10O2 | 2867559 | 0.637 |
| 4.126 | 2-Methylpropyl methyl ketone | 96 | 100 | C6H12O | 350687 | 0.078 |
| 4.309 | Ethylene glycol, formal | 96 | 74 | C3H6O2 | 143007 | 0.032 |
| 4.583 | Toluene | 94 | 92 | C7H8 | 64387 | 0.014 |
| 4.723 | 2-Methylpropyl acetate | 97 | 116 | C6H12O2 | 1732150 | 0.385 |
| 5.194 | Hexanal | 96 | 100 | C6H12O | 927859 | 0.206 |
| 6.323 | Ethylbenzene | 96 | 106 | C8H10 | 246876 | 0.055 |
| 6.896 | Styrene | 98 | 104 | C8H8 | 735459 | 0.163 |
| 7.138 | Ethylene glycol monoethyl ether | 90 | 90 | C4H10O2 | 18571 | 0.004 |
| 9.812 | 3-[1,3]dioxolan-2-ylpropyl ester | 93 | 174 | C8H14O4 | 51231 | 0.011 |
| 12.496 | 3-Buten-2-one | 90 | 88 | C5H12O | 4910 | 0.001 |
| 13.218 | 2,2-Dimethylbutane | 92 | 86 | C6H14 | 4315 | 0.001 |
|  | Others |  |  |  | 27411874 | 6.086 |
|  | Total |  |  |  | 450373540 | 100.000 |

Table S14. Chemical characterization of the volatile compounds profile in the reaction product obtained from of the test 2.1.5.

|  | **2.1.5** |  |  |  |  |  |
| --- | --- | --- | --- | --- | --- | --- |
| **TR** | **Compound** | **Similarity index (%)** | **MW g/mol** | **Molecular formula** | **Area** | **Area (%)** |
| 1.655 | Ethanol | 98 | 46 | C2H6O | 8217993 | 2.090 |
| 2.285 | Hexane | 96 | 86 | C6H14 | 186806 | 0.048 |
| 2.722 | Ethyl acetate | 84 | 88 | C4H8O2 | 299765213 | 76.236 |
| 3.362 | 1-Heptene | 97 | 98 | C7H14 | 330221 | 0.084 |
| 3.667 | Ethyl propanoate | 98 | 102 | C5H10O2 | 1490318 | 0.379 |
| 3.704 | Propyl acetate | 93 | 102 | C5H10O2 | 2249543 | 0.572 |
| 3.935 | Ethylidene diethyl ether | 92 | 118 | C6H14O2 | 329154 | 0.084 |
| 4.112 | Isopropylacetone | 95 | 100 | C6H12O | 270206 | 0.069 |
| 4.295 | Ethylene glycol, formal | 95 | 74 | C3H6O2 | 228265 | 0.058 |
| 4.714 | 2-Methyl-1-propyl acetate | 97 | 116 | C6H12O2 | 907459 | 0.231 |
| 5.192 | Hexanal | 95 | 100 | C6H12O | 4103608 | 1.044 |
| 6.323 | Ethylbenzene | 92 | 106 | C8H10 | 119989 | 0.031 |
| 6.493 | 1-Cyclohexene. 1-ethynyl- | 95 | 106 | C8H10 | 29025 | 0.007 |
| 7.006 | 2,3,3-Trimethylcyclobutanone | 94 | 112 | C7H12O | 1889 | 0.000 |
| 7.129 | Ethylene glycol ethyl ether acetate | 92 | 132 | C6H12O3 | 59807 | 0.015 |
| 7.334 | Ethylene glycol, formal | 97 | 74 | C3H6O2 | 4003 | 0.001 |
| 8.087 | (E)-2-Heptenal | 90 | 112 | C7H12O | 62070 | 0.016 |
| 9.336 | Propionic anhydride | 97 | 130 | C6H10O3 | 4747 | 0.001 |
| 9.813 | 3-Methoxy-3-methyl-2-butanone | 94 | 116 | C6H12O2 | 44745 | 0.011 |
|  | Other |  |  |  | 74803062 | 19.024 |
|  | Total |  |  |  | 393208123 | 100.000 |

Table S16. Chemical characterization of the volatile compounds profile in the reaction product obtained from of the test 2.2.1.

|  | **2.2.1** |  |  |  |  |  |
| --- | --- | --- | --- | --- | --- | --- |
| **TR** | **Compound** | **Similarity index (%)** | **MW g/mol** | **Molecular formula** | **Area** | **Area (%)** |
| 1.657 | Ethanol | 99 | 46 | C2H6O | 746768 | 0.370 |
| 2.578 | Ethyl acetate | 83 | 88 | C4H8O2 | 145808397 | 72.309 |
| 3.317 | Propylcyclopropane | 91 | 84 | C6H12 | 115360 | 0.057 |
| 3.627 | Ethyl propanoate | 94 | 102 | C5H10O2 | 72878 | 0.036 |
| 3.904 | 1,1-Diethoxyethane | 94 | 118 | C6H14O2 | 60900 | 0.030 |
| 8.738 | Isopropylcyclobutane | 91 | 98 | C7H14 | 9007 | 0.004 |
| 10.615 | Butylcyclopropane | 90 | 98 | C7H14 | 5163 | 0.003 |
| 10.986 | 3-Pentyne-2-one | 95 | 82 | C5H6O | 17044 | 0.008 |
|  | Others |  |  |  | 54811062 | 27.182 |
|  | Total |  |  |  | 201646579 | 100.000 |

Table 17. Chemical characterization of the volatile compounds profile in the reaction product obtained from of the test 2.2.2.

|  | **2.2.2** |  |  |  |  |  |
| --- | --- | --- | --- | --- | --- | --- |
| **TR** | **Compound** | **Similarity index (%)** | **MW g/mol** | **Molecular formula** | **Area** | **Area (%)** |
| 1.672 | Ethanol | 98 | 46 | C2H6O | 1293626 | 0.675 |
| 2.618 | Ethyl Acetate | 84 | 88 | C4H8O2 | 175737889 | 91.665 |
| 2.839 | 2-Methyl-1,3-dioxolane | 98 | 88 | C4H8O2 | 117918 | 0.062 |
| 3.641 | Ethyl propanoate | 99 | 102 | C5H10O2 | 743344 | 0.388 |
| 4.277 | Ethylene glycol, formal | 93 | 74 | C3H6O2 | 66631 | 0.035 |
| 4.559 | Toluene | 92 | 92 | C7H8 | 19827 | 0.010 |
| 4.693 | 2-Methylpropyl acetate | 97 | 116 | C6H12O2 | 588101 | 0.307 |
| 5.169 | Hexanal | 92 | 100 | C6H12O | 146704 | 0.077 |
| 6.309 | Ethylbenzene | 95 | 106 | C8H10 | 78793 | 0.041 |
| 6.480 | 1-Cyclohexene, 1-ethynyl- | 94 | 106 | C8H10 | 16823 | 0.009 |
| 6.884 | Styrene | 98 | 104 | C8H8 | 491788 | 0.257 |
| 7.124 | Methyl oxalate | 92 | 118 | C4H6O4 | 4888 | 0.003 |
| 7.736 | 1,3-Dioxolane-2-propanal, 2-methyl- | 95 | 144 | C7H12O3 | 13550 | 0.007 |
| 8.075 | 2-Heptenal | 90 | 112 | C7H12O | 25457 | 0.013 |
| 9.325 | 4,4-Dimethyl-1-pentene | 90 | 98 | C7H14 | 17343 | 0.009 |
| 9.804 | 3-Methoxy-3-methyl-2-butanone | 93 | 116 | C6H12O2 | 61929 | 0.032 |
| 10.532 | 2,2-Dimethylbutane | 91 | 86 | C6H14 | 9413 | 0.005 |
| 12.113 | 2,2,5,5-Tetramethylhexane | 93 | 142 | C10H22 | 4687 | 0.002 |
| 12.491 | 1-Pentanol | 95 | 88 | C5H12O | 12551 | 0.007 |
|  | Others |  |  |  | 12267304 | 6.399 |
|  | Total |  |  |  | 191718566 | 100.000 |

Table 18. Chemical characterization of the volatile compounds profile in the reaction product obtained from of the test 2.2.3.

|  | **2.2.3** |  |  |  |  |  |
| --- | --- | --- | --- | --- | --- | --- |
| **TR** | **Compound** | **Similarity index (%)** | **MW g/mol** | **Molecular formula** | **Area** | **Area (%)** |
| 1.652 | Ethanol | 98 | 46 | C2H6O | 1601920 | 0.781 |
| 2.608 | Ethyl acetate | 84 | 88 | C4H8O2 | 170347393 | 83.047 |
| 3.333 | 2-Propenal | 99 | 56 | C3H4O | 10001 | 0.005 |
| 3.445 | 1,2-Dineopentyldisulfane 1,1,2-trioxide | 97 | 254 | C10H22O3S2 | 18116 | 0.009 |
| 4.109 | Isopropylacetone | 92 | 100 | C6H12O | 48025 | 0.023 |
| 4.710 | 2-Methylpropyl acetate | 95 | 116 | C6H12O2 | 217501 | 0.106 |
| 6.898 | 1,3,5,7-Cyclooctatetraene | 93 | 104 | C8H8 | 106984 | 0.052 |
| 7.133 | Ethylene glycol monoethyl ether | 90 | 90 | C4H10O2 | 16015 | 0.008 |
|  | **Others** |  |  |  | **32755207** | **15.969** |
|  | **Total** |  |  |  | **205121162** | **100.000** |

Table S19. Chemical characterization of the volatile compounds profile in the reaction product obtained from of the test 2.2.4.

|  | **2.2.4** |  |  |  |  |  |
| --- | --- | --- | --- | --- | --- | --- |
| **TR** | **Compound** | **Similarity index (%)** | **MW g/mol** | **Molecular formula** | **Area** | **Area (%)** |
| 1.659 | Ethanol | 99 | 46 | C2H6O | 1565719 | 1.107 |
| 2.518 | Ethyl Acetate | 83 | 88 | C4H8O2 | 96092069 | 67.920 |
| 3.624 | Ethyl propanoate | 96 | 102 | C5H10O2 | 103926 | 0.073 |
| 4.078 | Butanedial | 92 | 86 | C4H6O2 | 9599 | 0.007 |
| 4.688 | 2-Methylpropyl acetate | 94 | 116 | C6H12O2 | 78451 | 0.055 |
|  | Others |  |  |  | 43628709 | 30.838 |
|  | Total |  |  |  | 141478473 | 100.000 |

Table S20. Chemical characterization of the volatile compounds profile in the reaction product obtained from of the test 2.2.5.

|  | **2.2.5** |  |  |  |  |  |
| --- | --- | --- | --- | --- | --- | --- |
| **TR** | **Compound** | **Similarity index (%)** | **MW g/mol** | **Molecular formula** | **Area** | **Area (%)** |
| 1.688 | Ethanol | 98 | 46 | C2H6O | 22899728 | 6.372 |
| 2.693 | Ethyl Acetate | 84 | 88 | C4H8O2 | 284002182 | 79.031 |
| 3.206 | 2.3-Dihydro-1,4-dioxine | 95 | 86 | C4H6O2 | 61481 | 0.017 |
| 3.349 | 1-Heptene | 92 | 98 | C7H14 | 57908 | 0.016 |
| 3.601 | 1.4-Dioxane | 97 | 88 | C4H8O2 | 79329 | 0.022 |
| 3.654 | Ethyl propanoate | 97 | 102 | C5H10O2 | 1388317 | 0.386 |
| 3.694 | Propyl acetate | 94 | 102 | C5H10O2 | 1545537 | 0.430 |
| 4.099 | 2-Methylpropyl methyl ketone | 96 | 100 | C6H12O | 213766 | 0.059 |
| 4.286 | Ethylene glycol, formal | 98 | 74 | C3H6O2 | 32453 | 0.009 |
| 4.566 | Toluene | 95 | 92 | C7H8 | 31252 | 0.009 |
| 4.701 | 2-Methylpropyl acetate | 98 | 116 | C6H12O2 | 979342 | 0.273 |
| 5.004 | 1-Octene | 93 | 112 | C8H16 | 89848 | 0.025 |
| 5.175 | Hexanal | 92 | 100 | C6H12O | 274647 | 0.076 |
| 6.314 | Ethylbenzene | 95 | 106 | C8H10 | 141313 | 0.039 |
| 6.606 | 3-Butene-2-one | 93 | 70 | C4H6O | 12675 | 0.004 |
| 6.886 | 1,3,5,7-Cyclooctatetraene | 97 | 104 | C8H8 | 652824 | 0.182 |
| 7.119 | Ethyl glycol acetate | 97 | 132 | C6H12O3 | 105840 | 0.029 |
| 9.808 | Ethylene glycol, formal | 95 | 74 | C3H6O2 | 56258 | 0.016 |
|  | **Others** |  |  |  | **46732181** | **13.004** |
|  | **Total** |  |  |  | **359356881** | **100.000** |
